# FABP7-mediated lipid-laden macrophages drive the formation of pre-metastatic niche and liver metastasis

**DOI:** 10.7150/ijbs.110750

**Published:** 2025-06-23

**Authors:** Shaowan Xu, Xin Peng, Zhenfang Wang, Chenchen Le, Xiangkun Wu, Zhicheng Zeng, Sisi Zeng, Ceng Zhang, Mingxing Qiu, Xin Zou, Hongxia Zhang, Feifei Wang, Wei Kang, Yanqing Ding, Li Liang

**Affiliations:** 1 Department of Pathology, Nanfang Hospital and School of Basic Medical Sciences, Southern Medical University, Guangzhou, China.; 2 Guangdong Provincial Key Laboratory of Molecular Tumor Pathology, Guangzhou, China.; 3 Jinfeng Laboratory, Chongqing, China; 4 Department of Pathology, The Fifth Affiliated Hospital, Sun Yat-sen University, Zhuhai, China.; 5 Department of Anatomical and Cellular Pathology, State Key Laboratory of Translational Oncology, Institute of Digestive Disease, State Key Laboratory of Digestive Disease, Prince of Wales Hospital, The Chinese University of Hong Kong, Hong Kong Special Administrative Region of China.

**Keywords:** pre-metastatic niche, fatty acid binding protein 7, macrophage, metastasis, metabolic reprogramming

## Abstract

Abnormal metabolism processes play a crucial role in the establishment of the pre-metastatic niche (PMN) and the subsequent metastasis to distant organs. However, the precise mechanisms underlying the lipid metabolic reprogramming of macrophages within the liver PMN remain elusive. In this study, we observed an upregulation of fatty acid-binding protein 7 (FABP7) in liver macrophages, which resulted in the accumulation of lipid droplets (LDs) within the PMN of colorectal cancer and pancreatic ductal adenocarcinoma. This accumulation was found to be mediated by the HIF-1α-induced expression of FABP7, which in turn enhanced DGAT1 activity in these macrophages. Furthermore, FABP7-induced lipid-laden macrophages were observed to deliver lipids to CD8^+^ T and tumor cells via exosomes. This process led to CD8^+^ T cell dysfunction and increased tumor cell proliferation through metabolic reprogramming. Importantly, genetic knockout or pharmacological inhibition of FABP7 reduced liver metastasis. Our findings reveal a novel mechanism involving FABP7-mediated LD in macrophages that contributes to liver PMN formation and metastasis. This suggests that targeting FABP7 may offer prognostic and therapeutic potential in addressing liver metastasis.

## Introduction

Metastasis is the main cause of cancer-associated mortality (>90%)^1^. The liver is the most common site of metastasis in gastrointestinal cancers, including colorectal cancer (CRC), pancreatic ductal adenocarcinoma (PDAC), and gastric cancer^2^. Primary tumors create a favorable microenvironment for subsequent tumor cell colonization in secondary organs through pre-metastatic niche (PMN) formation^3^. Liver metastasis of cancers depends on PMN formation and the pro-metastatic microenvironment^4^-^6^. Within the liver PMN, macrophages (including resident Kupffer cells (KCs) and monocyte-derived macrophages (MoMFs)) play pivotal roles in establishing pro-metastatic conditions^7^. Recent evidence indicates that liver KCs have been found to uptake integrin alphavbeta5 (αvβ5) from PDAC-derived exosomes and release the pro-inflammatory factor S100A8, leading to liver metastasis^8^. Cancer-derived exosomal HSPC111 promotes PMN formation and CRC liver metastasis by reprogramming the lipid metabolism in cancer-associated fibroblasts^9^. These findings suggest that targeting metabolic reprogramming in the PMN may support improved cancer interventions that directly treat tumor metastasis. However, the role of liver macrophage-mediated reprogramming of lipid metabolism in the PMN remains largely unknown.

Fatty acid-binding protein 7 (FABP7), also known as brain-type FABP (B-FABP) or brain lipid-binding protein (BLBP), is a member of the lipid-binding protein superfamily. As an intracellular lipid chaperone, FABP7 preferentially binds n-3 and n-9 polyunsaturated fatty acids (PUFAs) and participates in various cellular functions by controlling their uptake, metabolism, and intracellular storage^10^. FABP7 controls lipid raft function by regulating caveolin-1 expression, which mediates astrocytic responses to external stimuli^11^. In the nucleus, FABP7 can transmit its fatty acid ligand to activate PPARγ, which in turn regulates the transcription of lipid metabolism-related genes^12^. FABP7 is involved in the occurrence and development of various tumors and can serve as a prognostic marker. It is highly expressed in malignant glioma, breast cancer, and renal cell carcinoma, promotes cancer cell proliferation, migration, and invasion, and is associated with poor prognosis^13^, ^14^. However, the functions and molecular mechanisms of FABP7 in the tumor microenvironment (TME) have not yet been reported.

Abnormalities in lipid metabolism can confer a survival advantage to tumors, enhancing resistance to chemotherapy and radiation treatments, and alleviating cellular stress involved in the metastatic cascade^15^, ^16^. High CD36 expression promotes HCC development by triggering lipid metabolism reprogramming^17^. Lipid droplet (LD) accumulation has been increasingly recognized as a prominent characteristic of various cancers. LDs are common lipid storage organelles in eukaryotic cells that play an important role in maintaining the stability of the intracellular environment. Increased LD content can expand the sources of lipid substrates and energy, which can enable cancer cells to undergo metastatic cloning, especially in the TME^18^. LDs accumulation mediates the proliferation, invasion, metastasis, and chemoresistance of various cancers^19^-^21^. Notably, lung mesenchymal cells have been found to transfer LDs to cancer cells through exosomes and subsequently promote breast cancer metastasis^22^. However, the mechanism through which LDs accumulate in the liver PMN remains unclear.

In this study, we found that primary tumors induced FABP7 expression in liver macrophages via HIF-1α, leading to the accumulation of LDs by activating DGAT1 in the liver PMN. LDs accumulation in macrophages increased fatty acid oxidation (FAO), and induced the polarization of macrophages to the M2 type. Furthermore, LDs could be transported to CD8^+^ T cells and tumor cells through exosomes, thereby inducing CD8^+^ T cell dysfunction and tumor cell proliferation via metabolic reprogramming. Most importantly, pharmacological inhibition or knockout of FABP7 alleviated liver metastasis of cancers.

## Materials and Methods

### Human samples

The paraffin-embedded blocks of the human CRC and PDAC tissues were obtained from the Nanfang Hospital, Southern Medical University (Guangzhou, China), comprising 22 non-neoplastic specimens from patients with traumatic rupture or chronic cholecystitis (cohort 1, control), 26 liver cyst tissues with CRC (cohort 2, non-met), 30 CRC tissues with liver metastasis (cohort 3, metastatic), and 15 PDAC tissues with liver metastasis (cohort 4, metastatic). The clinical information of these samples was showed in Table S1. The study was approved by the Ethics Committee of the Nanfang Hospital of Southern Medical University (NFEC-2023-499).

### Mice

C57BL/6 mice were obtained from Medical Experimental Animal Center of Guangdong Province. FABP7 knockout (FABP7^-/-^) mice on a C57BL/6 J background were generated by Cyagen Biosciences Inc. (Suzhou, Jiangsu, China). Genotyping confirmed the FABP7^-/-^ mice through tail genomic DNA analysis (Figure S3B), mRNA expression profiling (Figure S3C) and protein quantification (Figure S3D). The genotyping primer sequences are provided in Table S3. CD45.1 mice were generously donated by Zhizhang Wang (Southern Medical University). All experiments used male mice aged six to eight weeks. The Institutional Animal Care and Use Committee of Southern Medical University approved all animal procedures (SMUL202409019).

### Cell culture and treatment

The MC38, PAN02, RAW264.7 and 293T cell lines were sourced from American Type Culture Collection (ATCC). These cells were cultured in Dulbecco's modified Eagle's medium (DMEM, Gibco) supplemented with 10% fetal bovine serum (FBS, Gibco) and 1% penicillin/streptomycin, then incubated at 37 °C and 5% CO_2_. Hypoxic conditions were induced by treating with 200 μM CoCL_2_ for 24 h or 1% O_2_ for 48 h before total RNA extraction. Lentiviral transduction was used to generate MC38 and PAN02 cells stably expressing luciferase, as well as GFP. Lentiviral transfection established RAW264.7 cells and bone marrow-derived macrophages (BMDMs) overexpressing FABP7. Cells were treated with SBFI-26 (200 nM, MedChemExpress), DHA (50 μM, MedChemExpress), the DGAT1 inhibitor A922500 (100 μM, Selleck), or GW4869 (20 μM, Selleck). Table S4 provides complete reagent details.

### Liver metastasis model

In the intrasplenic injection model of hepatic tumor metastasis, mice received either 5×10^5^ MC38 CRC cells or 1×10^6^ PAN02 PDAC cells suspended in 100 µl PBS, with 100 µl PBS alone serving as control^23^. For orthotropic CRC metastasis studies, we injected 1×10^6^ MC38 cells into the mesenteric border near the cecal tip in C57BL/6 mice^24^. Livers specimens were subsequently excised and analyzed by counting metastatic nodules and area to determine tumor burden.

In some experiments, macrophages and CD8^+^ T cells were depleted by intraperitoneal (i.p.) injection of clodronate liposomes (200 µl/animal, administered every two days) and anti-CD8α (200 µg/animal, twice weekly), respectively. Mice also received i.p. injections of SBFI-26 (20 mg/kg, daily) to inhibit FABP7, oral doses of A922500 (3 mg/kg, daily) to suppress DGAT1 and i.p. injections of GW4869 (1.25 mg/kg, daily) to block exosomes activity.

### Preparation of BMDMs

Bone marrow cells were isolated from the tibiae and femurs of 6-8-week-old mice and differentiated in DMEM growth medium supplemented with 10 ng/mL GM-CSF (PeproTech). Macrophages were harvested between days 6 and 8 for subsequent experiments. For transplantation, bone marrow-derived macrophages (BMDMs) were isolated from CD45.1 mice aged 6-8 weeks and injected intravenously (2×10^6^ cells /mouse) into CD45.2 recipients on day 7. Transwell co-culture systems were established by seeding 5×10^5^ BMDMs in the upper chamber and 1×10^6^ MC38 tumor cells in the lower chamber. After 24 h of co-culture, both cell populations were collected for analysis. In some experiments, differentiated BMDMs were polarized into M1 phenotypes using LPS (100 ng/ml, MedChemExpress) and IFN-γ (10 ng/mL, PeproTech), while M2 polarization was induced with IL-4 (20 ng/mL, PeproTech) and IL-10 (20 ng/mL, PeproTech) for 24 h.

### Cell isolation

To isolate liver macrophages, the liver was perfused in situ via the portal vein with Hank's buffered salt solution (HBSS, Gibco) containing 10 mM EDTA at 37 °C for 15 min. This was followed by perfusion with DMEM supplemented with 0.5 mg/ml collagenase IV (Sigma) under the same conditions. The digested liver was excised, and the resulting suspension was filtered through a 40 μm nylon mesh. After two centrifugation steps at 50 g (4 °C, 2 min each), the supernatant underwent further centrifugation at 300g for 5 min, and the pellet was resuspended in buffer. Liver macrophages were isolated using a mouse F4/80^+^ positive selection kit (STEMCELL), with immunofluorescence (IF) confirming >90% purity of the purified macrophages (Figure S2C).

To isolate CD8^+^ T cells, single-cell suspensions from murine spleens were prepared through mechanical disruption in PBS. After centrifugation at 300 g for 10min, CD8^+^ T cells were isolated using a mouse-specific magnetic bead isolation kit (STEMCELL). The purified cells were resuspended in culture medium and plated at 5×10^5^ cells per well in 24-well plates.

To isolate tumor infiltrating CD8^+^ T cells (TILs), metastatic liver tumor niches were dissociated using RPMI 1640 medium supplemented with 1 mg/ml collagenase IV (Sigma), 0.5 mg/ml DNase I (Sigma), and 0.5 mg/ml hyaluronidase (Sigma) at 37 °C for 45-60 min. The digested cell suspension was filtered through a 40-μm nylon mesh and centrifuged at 300 g for 5 min. Flow cytometry was used to quantify the resulting single-cell suspensions.

### Bioinformatics analysis​

Public gene expression data were obtained from the Gene Expression Omnibus (GEO, https://www.ncbi.nlm.nih.gov/geo/). The GSE156429 dataset (n=6) compared liver Kupffer cells between control mice and colorectal cancer (CRC) metastasis-bearing models, with limma-normalized microarray data analyzed for differentially expressed genes (FDR<0.05, |log₂FC|>1). The GSE109480 dataset (n=10) evaluated pre-metastatic liver tissues in Kras/p53-driven pancreatic cancer models using Wilcoxon rank-sum tests on FPKM-normalized RNA-seq data (raw *p*<0.05, |Δlog₂|>1).

The Kaplan-Meier Plotter database (https://kmplot.com)^25^ assessed survival correlations using mRNA microarray data from 1,061 colon cancer patients for overall survival (OS) analysis and 1,336 patients for relapse-free survival (RFS) analysis. Maximum-rank statistics automatically determined optimal cutoff values, with log-rank p-values <0.05 considered significant. Patients were stratified into high- and low-FABP7 expression groups based on these cutoffs: 8 (expression range 0-187) for OS and 3 (range 0-162) for RFS. Cox regression analysis calculated hazard ratios (HR).

All statistical analyses used R (v4.2.3) with the survival (v3.5-5) and ggplot2 (v3.4.2) packages.

### RNA sequencing (RNA-seq)

For RNA-seq analysis of liver macrophages, macrophages were isolated from WT and FABP7^-/-^ mice bearing MC38 tumors, washed with PBS, and lysed in Trizol reagent. The Genome Technologies core facility at Genedenovo Biotechnology Co., Ltd (Guangzhou, China) performed RNA extraction, quality assessment, and library preparation. Sequencing of cDNA libraries was carried out on the Illumina platform by the same facility. These data are available in the Gene Expression Omnibus under accession number GSE244156.

### Flow cytometry and cell sorting

To characterize liver macrophage phenotypes and FABP7 expression, the liver tissues from naive control and tumor-bearing mice were collected and the single-cell suspensions were prepared as described above. Surface markers (CD45, F4/80, CLEC4F, CX3CR1, CD163, CD68, CD45.1, CD45.2, CD86 and CD80) were stained with fluorescent antibodies at 4 °C for 30 min^23^. For intracellular FABP7 detection, cells were fixed and permeabilized using the BD Biosciences Kit, then incubated with anti-FABP7 antibody at 4 °C for 30 min. After washing, cells were labeled with fluorescent secondary antibodies for 30 min at 4 °C before flow cytometry analysis^26^. Complete antibody specifications appear in Table S4.

To assess hepatic macrophage lipid content, we prepared cell suspensions following the established protocol. The cells were stained with 0.2 µg/ml BODIPY 493/503 at 37 °C for 30 min under dark conditions, followed by flow cytometric quantification.

To investigate LD transfer from macrophages to tumor cells or CD8^+^ T cells, we labeled macrophages with 1 mM BODIPY FL C16 for 30 min at 37 °C. After five washes to eliminate extracellular BODIPY, the cells were cultured in DMEM, and condition medium (CM) was collected 24 h later. The culture system contained 5×10^5^ BMDMs, 5×10^5^ MC38 cells, or 5×10^5^ CD8^+^ T cells. Following 24-hour coculture with labeled BMDMs or exposure to CM, BODIPY FL C16 fluorescence in recipient cells were quantified by flow cytometry and macrophage viability was assessed with a LIVE/DEAD Cell Stain Kit^22^, ^27^.

To assess the specificity of CD8^+^ T cells isolated from FABP7 KO mice, TIL-CD8^+^ T cell suspensions were prepared as described above. After incubating with FcR blocking reagent for 5 min at room temperature, cells were stained with H-2Kb OVA Tetramer-SIINFEKL-APC for 30-60 min at 4 °C in the dark. For intracellular cytokine detection of interferon-γ (IFN-γ), granzyme B (GzmB) or tumor necrosis factor-α (TNF-α) in CD8^+^ T cells, surface staining with anti-CD45 and anti-CD8a antibodies proceeded for 30 min at 4 °C. Intracellular staining was performed by using the Fixation/Permeabilization Solution Kit (BD Biosciences), followed by for 30 min at 4 °C with anti-IFNγ, anti-GZMB or anti-TNF-α antibodies before flow cytometry analysis. All antibodies were diluted 1:200^28^.

### T cell stimulation assay

CD8^+^ T cells isolated from splenocytes were cultured with BMDMs-CM supplemented with 5 µg/ml anti-CD3 and 2 µg/ml anti-CD28 antibodies. Following 24-hour incubation, the cells were harvested and analyzed by flow cytometry to quantify GzmB, TNF-α and IFN-γ expression levels^28^.

### Cellular energy metabolism analysis

To measure the oxygen consumption rate (OCR) in tumor cells, MC38 cells were pre-incubated with CM from Bodipy-C16-labeled BMDMs. Liver macrophage OCR was measured using macrophages isolated from WT and FABP7^-/-^ mice bearing MC38 tumors. For simultaneous OCR and extracellular acidification rate (ECAR) measurements in CD8^+^ T cells, activated cells were treated with CM from Bodipy-C16-labeled BMDMs. All assays were performed in 96-well Seahorse plates seeded with 2×10^4^ macrophages, 1×10^4^ MC38 cells, or 2×10^4^ CD8^+^ T cells.

For Cell Mitochondrial Stress Test, mitochondrial stress was evaluated using the Seahorse Bioscience protocol (Agilent, US). Macrophages, MC38 cells, and activated CD8^+^ T cells were cultured in Seahorse XF DMEM assay medium containing 1 mM pyruvate, 2 mM glutamine, and 10 mM glucose. OCR measurements followed sequential administration of oligomycin (Oligo, 1.5 µM), fluoro-carbonyl cyanide phenylhydrazone (FCCP, 1 µM), and Rotenone/Antimycin (Rot/Ant, 0.5 µM). Tumor cells received either vehicle or 25 mM etomoxir 15 min before plate loading. Basal and Max ΔOCR values represented respiration changes with or without etomoxir treatment, calculated as specified by the manufacturer. Data normalization to cell counts and processed using XF Wave software^27^.

For Glycolysis Stress Test, CD8^+^ T cells were cultured in Seahorse XF assay Medium containing 2mM glutamine. ECAR measurements reflected cellular responses to sequential treatment with glucose (10 mM), Oligo (1 mM), and 2-deoxy-D-glucose (2-DG, 50 mM). Data normalization to cell counts and processed using XF Wave software^22^.

The mitochondrial fuel flex test evaluated cellular dependency on glucose, glutamine, and LCFAs as oxidative substrates according to the manufacturer's protocol. Sequential treatment with BPTES (3 µM), etomoxir (Eto, 4 µM), and UK5099 (2 µM) preceded OCR measurements before and after each inhibitor. Data normalization to cell counts and processed using XF Wave software.

For Palmitate Oxidation Stress Test, mitochondrial stress was measured following the manufacturer's protocol, with cells assayed in Seahorse XF assay Medium containing 2 mM glucose, 0.5 mM L-carnitine, and either 1 mM palmitate-BSA or BSA Control. OCR measurements reflected the sequential addition of etomoxir (4 μM), Oligo (1.5 μM), FCCP (1 μM), and Rot/Ant (0.5 μM). Data normalization to cell counts and processed using XF Wave software.

### MS analysis of ^13^C metabolites

For [1-^13^C_1_] acetate tracing, 5× 10^6^ BMDMs were cultured for 24 h in medium containing 10% FBS, 0.25 mM sodium pyruvate, 1 mM L-glutamine, and either 0.5 mM D-glucose (control) or 2 nM [1-^13^C_1_] acetate (MedChemExpress). Parallel experiments with [^13^C_16_] palmitate employed identical cell numbers cultured for 12 h in control medium or medium supplemented with 100 μM [^13^C_16_] palmitate (MedChemExpress) 29. Metabo-Profile Biotechnology (Shanghai) Co., Ltd performed all sample pretreatment and quality control procedures. Metabolite derivatives were analyzed using an ACQUITY-I UPLC /Xevo TQS triple quadrupole mass spectrometer. Waters'MassLynx software (v4.1) processed the raw UPLC-MS data for metabolite peak extraction, integration, identification, and quantification. Further statistical analysis was performed using R language (v4.1.1).

### Histology staining

For immunohistochemistry (IHC) analysis of liver metastatic colonization, liver tissues were fixed in 10% formalin and paraffin-embedded. The blocks were sectioned at 2.5 μm and mounted on glass slides. After treatment with 3% hydrogen peroxide, sections were incubated overnight at 4°C with primary antibodies diluted 1:200. For immunofluorescence (IF), sections were stained for 1h at room temperature with goat anti-mouse or anti-rabbit IgG/Alexa Fluor (Bioss Antibodies), followed by 5 min DAPI nuclear counterstaining. IHC staining employed goat anti-mouse or anti-rabbit IgG (ZSGB-BIO Antibodies) under identical incubation conditions, with DAPI counterstaining performed equivalently.

To quantify lipid levels in isolated liver macrophages, *ex vivo* or *in vitro* cultured cells were stained with 2 μM BODIPY 493/503 for 30 min at 37 °C, followed by fixation with 4% PFA and nuclear counterstaining with DAPI.

For assessing lipid transfer from macrophages to tumor cells or CD8^+^ T cells, macrophages were pre-labeled with BODIPY FL C16 (1 mM) for 30 min at 37 °C before co-culturing with MC38 cells and CD8^+^ T cells or culturing with CM, after which samples were fixed with 4% PFA and nuclei were stained with DAPI.

To examine lipid-mitochondria colocalization, MC38 cells were adhered to glass slides via Cytospin, then incubated with CM from BODIPY FL C16-labeled BMDMs, stained with MitoTracker® Red CMXRos (200 nM) for 30 min at room temperature, fixed with 4% PFA and finally counterstained with DAPI for 5 min at room temperature.

### Transmission electron microscope (TEM)

Liver macrophages were isolated as previously described and fixed by immersion in 2.5% glutaraldehyde for 1 h at room temperature, followed by overnight fixation at 4 °C. After removing the fixation, the cells were rinsed with cold PBS. Gradient acetone dehydration preceded embedding in Spurr epoxy resin for polymerization. Ultrathin sections (50 nM) were cut using a Leica EM UC7 ultramicrotome and double-stained with uranyl acetate and lead citrate for TEM observation (Hitachi H-7500, Japan).

### Western blot

Tissue and cells lysates were prepared using RIPA buffer (Fdbio science) and protein concentrations were determined with the Bradford Protein Assay (KeyGEN BioTECH). Protein extracts of equal quantity underwent SDS-PAGE separation before electroblotting onto nitrocellulose membranes (Millipore). The membranes were probed with primary antibodies (1:1000 dilution) at 4 °C overnight. After incubation with HRP-conjugated secondary antibody (Fdbio Science, 1:10000 dilution), protein bands were visualized using FDbio-Femto ECL Western blotting detection reagents (Fdbio Science). ImageJ software quantified protein expression levels relative to β-actin.

### Quantitative real-time PCR (qPCR)

Total RNA was isolated with TRIzol reagent (Takara) and reverse transcribed into cDNA. SYBR Green PCR Master Mix (Takara) served as the detection system for qPCR. Gene expression levels were quantified via the 2-ΔΔCt method, with β-actin as the endogenous control. Primers sequences are provided in Table S3.

### CHIP

Following the manufacturer's protocol (#9003, CST), BMDMs were fixed with 37% formaldehyde and quenched with glycine before chromatin digestion. The lysate was incubated with HIF-1α antibody, followed by immunoprecipitation using ChIP-grade Protein G magnetic beads to isolate the target protein DNA complex. Purified DNA was subsequently analyzed by qPCR.

### Luciferase reporter assay

293T cells were transfected with HIF-1α and/or the pGL4.10-mFABP7 promoter plasmid (Youming Biotechnology Co., Ltd, Guangzhou, China). Luciferase reporter assays were subsequently performed on the harvested cells following the manufacturer's protocol (#FR201, Transgen).

### Exosome isolation and detection

BMDMs were maintained in DMEM supplemented with 10% FBS and subjected to ultracentrifugation at 120,000 g for 8 h at 4 °C before processing. The CM was collected and centrifuged to eliminate cellular debris. Exosomes isolation involved sequential centrifugation steps: 300 g for 10 min, 2000 g for 10 min, and 10,000 g for 30 min at 4 °C, culminating in ultracentrifugation at 120,000 g for 70 min. The purified exosomes were washed with PBS and resuspended in fresh PBS. After lysing the pellets in RIPA buffer, proteins (20 μg) were separated by western blot and probed with anti-CD63 (1:500, ABClonal A5271) and anti-TSG101 (1:1000, Immunoway B6001).

Nanoparticle tracking analysis (NTA) was performed with a ZetaView instrument (Partical Metrix, Germany) to determine the concentration and size distribution of both CM and isolated exosomes. The CM was diluted 1:50 dilution in pure water, while exosomes required a 1:1000 dilution prior to measurement. Each analysis used 1 mL of prepared sample, with data collection and processing conducted through the ZetaView software. Comparative evaluation focused exclusively on exosome counts and concentrations within the 80-180 nm size range.

PKH26 labeling of BMDMs or exosomes involved incubation with the dye (1:250 dilution) for 15 min at 37 °C and followed by 30 min at 4 °C. After staining, BMDMs were washed with PBS and centrifuged at 950 rpm for 5 min, while exosomes underwent PBS washing and ultracentrifugation at 120,000 g for 70 min at 4 °C. For cellular uptake experiments, 5 µg of exosomes were incubated with 5×10^5^ recipient cells for 24 h. To track exosome distribution *in vivo*, mice received 150 µg of exosomes per animal via tail vein injection.

### Measurement compositions of the CM

Liver macrophages isolated from naive control or FABP7^-/-^ mice bearing MC38 cells cultured *ex vivo* to analyze their CM composition. Lipid content was quantified using the Triglyceride Colorimetric Assay Kit and Free Fatty Acid Fluorometric Assay Kit (Cayman Chemical) according to the manufacturer's protocols.

### Statistical analysis

The data are presented as mean ± SEM. All statistical analyses were conducted in GraphPad Prism 6, with unpaired two-tailed Student's t-test applied for two-group comparisons and one-way or two-way ANOVA for multi-group analyses. P < 0.05 was considered statistically significant, with the following notation: * p < 0.05, ** p < 0.01, *** p < 0.001, **** p < 0.0001, and ns (not significant, p > 0.05).

## Results

### FABP7 is upregulated in macrophages within the liver PMN

To investigate metabolic alterations in the liver microenvironment during progression from PMN formation to metastasis, we examined public gene expression profiles of liver KCs from tumor-free control mice and CRC metastasis models (GSE156429)^30^, along with liver tissues from control mice and pre-metastatic PDAC-bearing KPC mice (GSE109480)^31^. Intersecting these datasets with lipid metabolism pathway genes from the Reactome pathway database identified two differentially expressed genes (DEGs): FABP7 and aryl-hydrocarbon receptor repressor (AHRR). As FABP7 was the most significantly upregulated lipid metabolism-related gene in both datasets, we selected it for further investigation (Figure S1A-S1C).

**Figure 1:**
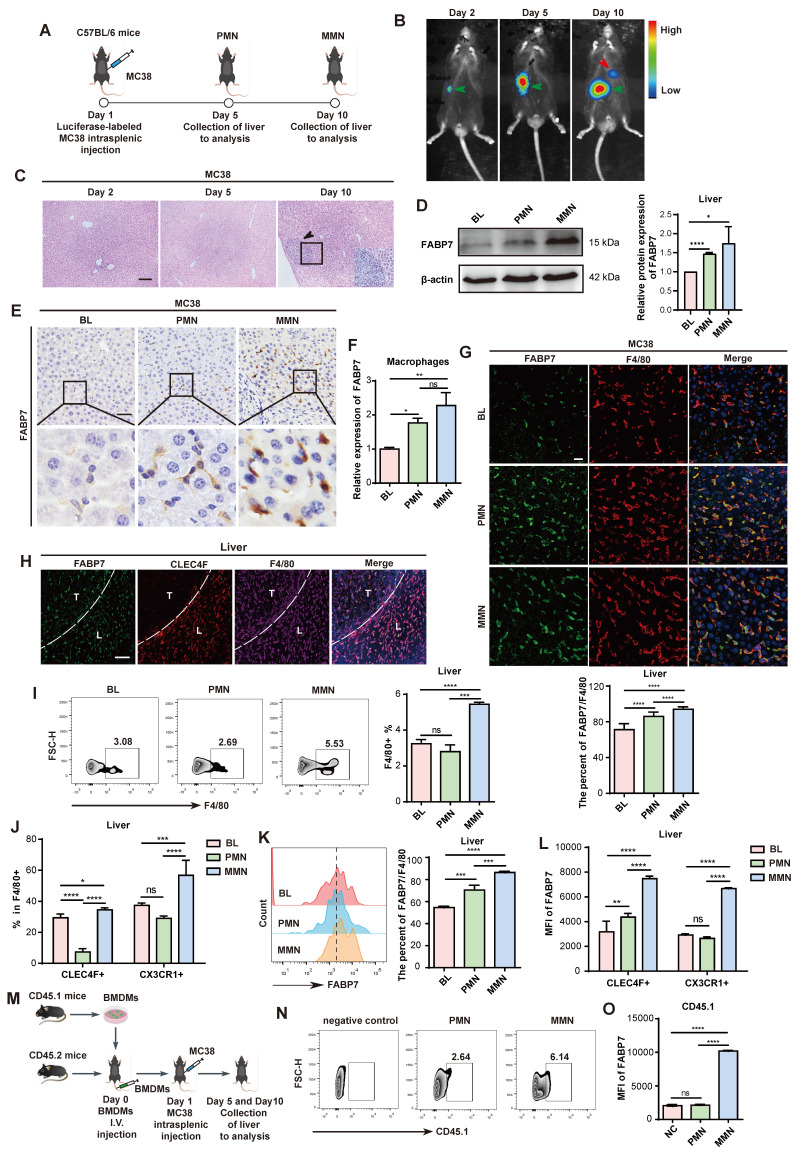
**FABP7 is upregulated in liver macrophages within the PMN stage.** (A) Schematic illustration of the intrasplenic injection model using MC38 cells to study liver metastasis (By Figdraw). (B) Representative bioluminescence images of luciferase-expressing MC38 cells following intrasplenic injection in C57BL/6 mice. The green arrow indicated the splenic tumor in situ, while the red arrow indicated the liver metastases. (C) HE staining of liver sections from MC38-injected mice at days 2, 5, and 10 post-injection. Scale bars, 100 μm. (D-E) Western blot analysis (D) and IHC staining (E) of FABP7 expression in livers from MC38 tumors bearing compared to tumor-free controls (BL) in PMN and MMN (n=3). Scale bars, 50 μm. β-actin served as loading control. (F) qPCR analysis of FABP7 expression in liver macrophages isolated from MC38 tumors bearing mice across three different stages (n=3). (G) IF staining of FABP7 (green) and F4/80 (red) in livers from MC38 tumors bearing mice at three different stages. The percentage of FABP7^+^ proportions within F4/80^+^ populations was shown below (n=3). Scale bars, 20 μm. (H) IF staining of FABP7 (green), CLEC4F (red) and F4/80 (purple) in livers from mice bearing MC38 cells for 10 d. T, metastatic tumor; L, adjacent liver. Scale bars, 50 μm. (I-L) Flow cytometry analysis of the number (I and J) and FABP7 expression (K and L) in total macrophages (F4/80^+^), KCs (CLEC4F^+^) or MoMFs (CX3CR1^+^) in livers at three different stages (n=3). (M) Pattern diagram of BMDMs adoptive transfer experiment. (N-O) Flow cytometry measured CD45.1^+^ macrophage count and FABP7 expression levels at three different stages (n=3). HE, hematoxylin-eosin; IHC, immunohistochemistry; IF, immunofluorescence. Data are presented as the mean ± SEM. p values were determined by one-way ANOVA (D, F, G, K and O) or two-way ANOVA (J and L).: * p < 0.05, ** p < 0.01, *** p < 0.001, **** p < 0.0001; ns, not significant.

To investigate changes in FABP7 expression from the liver PMN to the macro-metastatic niche (MMN), we employed an *in vivo* mouse model based on a previous study^32^. Intrasplenic injection of murine CRC (MC38) and PDAC (PAN02) cell lines reliably induced liver metastasis (Figure 1A; Figure S1D), monitored through bioluminescence imaging and histopathological analysis. Mice injected with MC38 cells developed liver PMNs by day 5 and MMNs by day 10, while PAN02-injected mice formed PMNs by day 14 and MMNs by day 21 (Figure 1B, 1C; Figure S1E, S1F).

Western blot analysis confirmed FABP7 upregulation in both the PMN and MMN stages of MC38 tumor-bearing mice (Figure 1D). qPCR and IHC analyses further demonstrated significantly higher FABP7 levels in the PMN and MMN compared to normal mouse liver tissue (Figure 1E; Figure S1G-S1I). We also established an orthotopic model by injecting MC38 cells directly into the mouse cecum to induce in situ liver metastasis (Figure S1J). This approach resulted in liver micrometastases at 4 weeks and macrometastases at 6 weeks (Figure S1K). Western blot analysis showed a progressive increase in FABP7 levels from week 1 to week 6 in these MC38-bearing mice (Figure S1L). Given the potential link between liver steatosis and worsened CRC metastasis-associated mortality^33^, we assessed steatosis status using Oil Red O staining in the PMN and MMN liver tissues from MC38-inoculated mice. No evidence of steatosis was detected in these tissues (Figure S1M).

Given the established role of FABP7 in liver KCs and its contribution to liver fibrosis progression^34^, we assessed FABP7 expression within distinct liver PMN cell populations using IF staining. FABP7 was highly expressed in macrophages (F4/80^+^) but was low detectable in other immune and stromal cell types, including neutrophils (Ly6G^+^), B cells (CD19^+^), T cells (CD4^+^ and CD8^+^), and fibroblasts (aSMA^+^) (Figure S2A). Flow cytometry analysis confirmed minimal FABP7 expression (<5% positivity) in all T cell subsets, including CD3+ populations and double-negative T cells (Figure S2B). We isolated macrophages from Blank (BL), PMN, and MMN liver tissues via flow cytometry, confirming >90% purity of the sorted populations (Figure S2C). qPCR analysis revealed significantly increased FABP7 expression in macrophages isolated from both PMN and MMN liver tissues compared to controls (Figure 1F). Furthermore, macrophages within liver PMN and MMN tissues from tumor-bearing mice exhibited substantially higher FABP7 expression than those from tumor-free mice (Figure 1G; Figure S2D). Notably, FABP7 expression was minimal to undetectable in macrophages from the lung, bone, or spleen—common metastatic sites for gastrointestinal tumors—during the PMN phase (Figure S2E).

We next examined FABP7 expression dynamics across distinct liver macrophage populations within the PMN and MMN. IF staining revealed FABP7 expression not only in KCs (CLEC4F^+^ F4/80^+^) but also in CLEC4F^-^ F4/80^+^ macrophages within the liver MMN (Figure 1H). To validate this observation, we employed flow cytometry to assess FABP7 expression in CLEC4F^+^ F4/80^+^ KCs and CX3CR1^+^ F4/80^+^ MoMFs from the liver PMN and MMN of MC38 tumor-bearing mice (Figure S2F). Macrophage abundance increased significantly in the MMN but not the PMN, with elevations observed in both KC and MoMF populations (Figure 1I, 1J). Our analysis of total F4/80^+^ macrophages (including KCs and MoMFs) showed that FABP7 expression gradually increased during the PMN and MMN stages (Figure 1K). Additionally, FABP7 elevation during the PMN phase was primarily confined to KCs, whereas both KCs and MoMFs showed increased FABP7 expression during MMN development (Figure 1L). To track the origin of MoMFs, we utilized a bone marrow-derived macrophage (BMDM) transplantation model (Figure 1M). Flow cytometry demonstrated an increased number of CD45.1^+^ BMDMs within both PMN and MMN liver tissues (Figure 1N). Importantly, FABP7 upregulation in these CD45.1^+^ BMDMs was specifically observed only in the liver MMN (Figure 1O). Collectively, these findings demonstrate that FABP7 expression is predominantly elevated in KCs during the liver PMN stage, while both KCs and recruited MoMFs exhibit upregulated FABP7 expression in the established MMN.

### FABP7 is a prognostic and therapeutic biomarker for tumor liver metastasis

**Figure 2:**
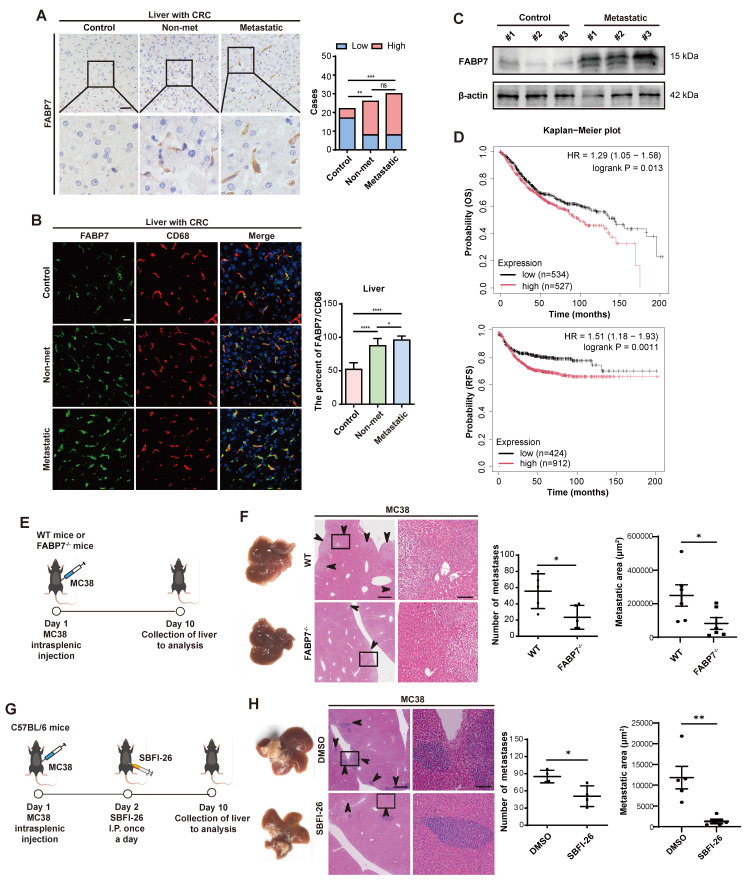
** FABP7 is a prognostic and therapeutic biomarker for liver metastasis.** (A) Representative IHC images of FABP7 expression in liver samples from CRC patients with liver metastatic (n=30), non-metastatic (n=26), and non-cancerous controls (n=20). Statistical analysis using chi-square test was shown on the right. Scale bar, 20 μm. (B) Representative IF images of FABP7 (green) and macrophage marker CD68 (red) in livers. The percentage of FABP7 to CD68 was shown on the right (n=10 per group). Scale bar, 20 μm. (C) Western blot analysis of FABP7 expression levels in liver samples from metastatic CRC patients compared with controls (n=3). β-actin served as loading control. (D) OS and RFS curves based on FABP7 expression level using mRNA microarray data of colon cancer from the Kaplan-Meier Plotter database. (E-F) As depicted in the schematic (E), the number and area of liver metastasized foci were compared between WT and FABP7^-/-^ mice bearing MC38 tumors (F, n=6). Scale bars, 200 μm (F, left of the middle panel) or 20 μm (F, right of the middle panel). (G-H) As depicted in the schematic (G), the effect of SBFI-26 in controlling liver metastasis was determined (H, n=5). Scale bars, 200 μm (H, left of the middle panel) or 20 μm (H, right of the middle panel). CRC, colorectal cancer; OS, overall survival; RFS, relapse free survival. Data are presented as mean ± SEM. p values were determined by chi-square test (A), one-way ANOVA (B) or unpaired two-tailed Student's t-test (F and H). * p < 0.05, ** p < 0.01, *** p < 0.001, **** p < 0.0001; ns, not significant.

We obtained liver tissues from 22 non-neoplastic control patients (cohort 1) diagnosed with traumatic rupture or chronic cholecystitis. Additionally, we collected liver cyst tissues from 26 CRC patients without metastasis (cohort 2, non-metastatic), and metastatic liver tissues from 30 CRC patients (cohort 3) and 15 PDAC patients (cohort 4). We then analyzed FABP7 expression across all cohorts. IHC and IF analyses demonstrated significantly elevated FABP7 levels in macrophages from both non-metastatic and metastatic liver tissues of CRC patients (Figure 2A and 2B). Similarly, PDAC patients with liver metastasis exhibited higher FABP7 expression (Figure S2G). Western blot analysis confirmed increased FABP7 expression in metastatic CRC and PDAC liver samples compared to controls (Figure 2C; Figure S2H). To assess the clinical relevance of FABP7, we performed survival analysis using colon cancer mRNA microarray data from the Kaplan-Meier Plotter database. Among 1,061 patients with overall survival (OS) data and 1,336 with relapse-free survival (RFS) data, elevated FABP7 expression was significantly associated with worse clinical outcomes. Specifically, patients with higher FABP7 expression exhibited reduced OS (HR = 1.29, 95% CI: 1.05-1.58, p = 0.013) and RFS (HR = 1.51, 95% CI: 1.18-1.93, p = 0.0011) compared to the low-expression group (Figure 2D).

To determine the functional contribution of FABP7 to liver metastasis, we generated systemic FABP7 knockout (KO) mice (FABP7^-/-^) and confirmed successful FABP7 ablation in liver macrophages (Figure S3A-S3D). Importantly, the knockout did not alter the M1 or M2 polarization states of KCs or MoMFs in the liver (Figure S3E-S3G), nor did it affect macrophage expression of antigen presentation markers (CD80 and CD86) under physiological homeostasis (Figure S3H). Similarly, macrophage M1/M2 phenotypes in lung and spleen tissues remained unchanged in FABP7 KO mice at homeostasis (Figure S3I). Finally, FABP7 KO did not reduce the number or impair the function of regulatory T cells (Tregs) in MC38 tumor-bearing mice (Figure S3J).

To evaluate the role of FABP7 in liver PMN formation and metastasis, we intrasplenically injected MC38 and PAN02 cells into FABP7^-/-^ mice and their wild-type (WT) littermates. Liver metastases were quantified 10 days (MC38) and 21 days (PAN02) post-inoculation (Figure 2E; Figure S3K). Both the number and area of liver metastasis foci were significantly reduced in FABP7^-/-^ mice compared to WT controls (Figure 2F; Figure S3L). To pharmacologically inhibit FABP7, we employed the specific inhibitor α-Truxillic acid 1-naphthyl ester (SBFI-26). This compound competitively binds the FABP7 ligand-binding site, blocking its biological function, and has shown efficacy in murine inflammatory pain models^35^, ^36^, although its impact on tumor associated macrophage remained unexplored. We administered SBFI-26 via daily intraperitoneally injected to MC38-inoculated C57BL/6 mice. Consistent with the genetic deletion results, SBFI-26 treatment dramatically reduced both the number and area of liver metastasis foci (Figure 2G and 2H). These findings establish macrophage FABP7 expression as a critical regulator of liver metastasis with potential prognostic and therapeutic value.

### FABP7 induces M2 polarization of macrophages in liver PMN

**Figure 3:**
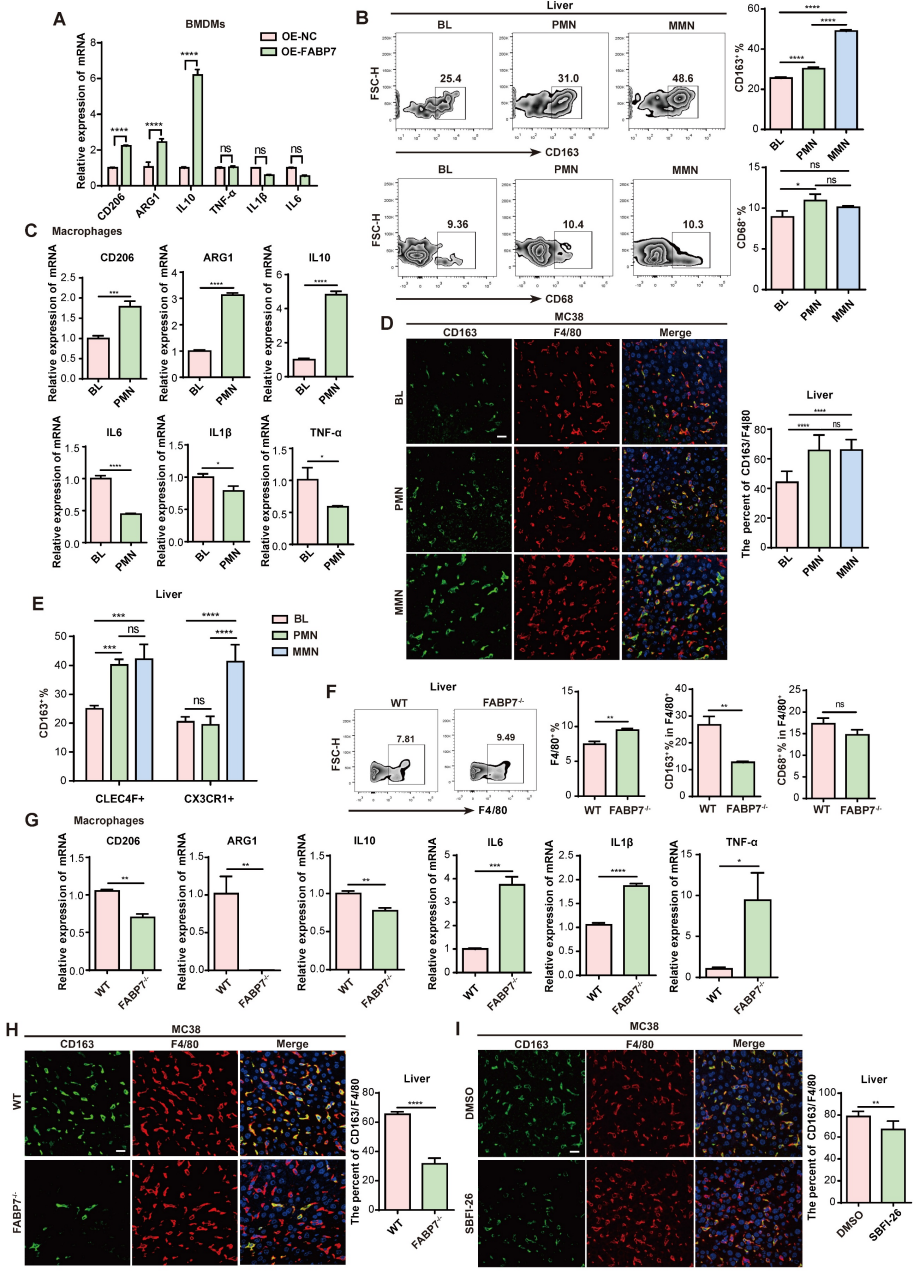
**FABP7 promotes M2 polarization of macrophage in liver PMN.** (A) qPCR analysis of M2-relative genes (CD206, ARG1, IL10) and M1-relative genes (TNF-α, IL1β, IL6) expression in OE-FABP7 BMDMs compared to the control cells (n=3). (B) Flow cytometry analysis of CD163^+^ M2 and CD68^+^ M1 macrophages in livers of MC38 tumor- bearing mice across three different stages (n=3). (C) qPCR analysis of M2 and M1 relative genes expression in liver macrophages isolated from tumor-free (BL) and MC38 tumor- bearing mice in PMN (n=3). (D) Representative IF images of CD163 (green) and F4/80 (red) in livers from mice at three different stages, with the CD163^+^/F4/80^+^ ratio quantified (n=3). Scale bars, 20 µm. (E) Flow cytometry analysis of CD163^+^ M2 macrophage populations in liver PMN and MMN (n=3). (F) Flow cytometry analysis of CD163^+^ and CD68^+^ expression levels in F4/80^+^ liver macrophages from WT and FABP7^-/-^ mice (n=3). (G) qPCR analysis of M2- and M1- relative genes expression in liver macrophages isolated from WT and FABP7^-/-^ mice in PMN (n=3). (H) Representative IF images of CD163 (green) and F4/80 (red) in livers from WT and FABP7^-/-^ mice in PMN, with the CD163^+^/F4/80^+^ ratio quantified (n=3). Scale bars, 20 µm. (I) Representative IF images of CD163 (green) and F4/80 (red) in livers from MC38 tumor-bearing C57BL/6 mice treated with DMSO or SBFI-26 in PMN, with the CD163^+^/F4/80^+^ ratio quantified (n=3). Scale bars, 20 µm. OE, overexpression. Data are presented as the mean ± SEM. p values were determined by two-way ANOVA (A and E), one-way ANOVA (B and D) or unpaired two-tailed Student's t-test (C and F-I). * p < 0.05, ** p < 0.01, *** p < 0.001, **** p < 0.0001; ns, not significant.

Macrophages represent functionally distinct subpopulations critical to immunity. To induce polarization, BMDMs were stimulated to an M1 (pro-inflammatory) phenotype with interferon-γ (IFN-γ) and lipopolysaccharide (LPS), or to an M2 (pro-tumoral) phenotype using interleukin-4 (IL-4) and IL-10^37^, ^38^. qPCR analysis confirmed M1-marker (TNF-α, IL1β, IL6) and M2-marker (CD206, ARG1, IL10) expression (Figure S4A). Assessment of FABP7 expression in M0 (unactivated), M1, and M2 BMDMs by qPCR revealed significant upregulation specifically in M2-polarized macrophages (Figure S4B). Investigating FABP7's influence on polarization, we found that its overexpression (OE) both in BMDMs and RAW264.7 cells significantly increased M2-marker expression, while leaving M1-marker levels largely unchanged (Figure 3A; Figure S4C-S4E). Flow cytometry demonstrated increased CD163^+^ M2 macrophage numbers in the liver PMN and MMN of MC38 tumor-bearing mice, contrasting with CD68^+^ M1 macrophages which showed no increase (Figure 3B). No polarization differences emerged between BL and PMN macrophages in lungs, bones, or spleens (Figure S4F). Further analysis of macrophages isolated from the liver PMN from MC38 tumor-bearing mice confirmed a significant increase in M2 markers and a concurrent decrease in M1 markers (Figure 3C). IF analysis validated this M2 enrichment, showing a significantly higher percentage of CD163^+^ F4/80^+^ M2 macrophages within both the PMN and MMN of mice bearing MC38 or PAN02 tumors (Figure 3D; Figure S4G). This pattern was recapitulated in human CRC tissues, where CD163^+^ CD68^+^ M2 macrophage infiltration increased, and CD86^+^ CD68^+^ M1 macrophage infiltration decreased, at both non-metastatic and metastatic stages (Figure S4H). Spatial analysis revealed CLEC4F^+^ KCs as the primary M2-expanding population during liver PMN formation, while both CLEC4F^+^ KCs and CX3CR1^+^ MoMFs contributed to M2 expansion in established metastases (Figure 3E).

To determine the functional role of FABP7 in macrophage conversion, we analyzed liver PMN macrophages from WT and FABP7^-/-^ mice bearing MC38 tumors and observed increased macrophage abundance in FABP7^-/-^ mice. FABP7 KO selectively reduced M2 macrophage infiltration without altering M1 macrophage levels (Figure 3F). qPCR analysis revealed that FABP7 KO downregulated M2 markers and upregulated M1 markers (Figure 3G). Consistent with this, IF revealed a reduced percentage of M2 macrophages in the liver PMN of FABP7^-/-^ versus WT mice (Figure 3H). *In vitro* studies confirmed this polarization shift: FABP7 KO inhibited IL-4/IL-10-driven M2 polarization in BMDMs but did not alter LPS/IFN-γ-induced M1 polarization (Figure S4I). Notably, this effect was liver-specific, as FABP7 KO increased splenic M2 macrophages in tumor-bearing mice (Figure S4J). Pharmacological inhibition using the FABP7-specific blocker SBFI-26 mirrored genetic ablation, markedly reducing M2 macrophage numbers in the liver PMN (Figure 3I; Figure S4K). qPCR analysis of isolated macrophages from SBFI-26-treated mice confirmed suppression of M2 markers and induction of M1 markers without altering FABP7 transcription (Figure S4L). Collectively, these data establish FABP7 as a critical driver of M2 macrophage polarization specifically within the liver PMN.

### FABP7 promotes LD accumulation by mediating DGAT1 in PMN liver macrophages

**Figure 4:**
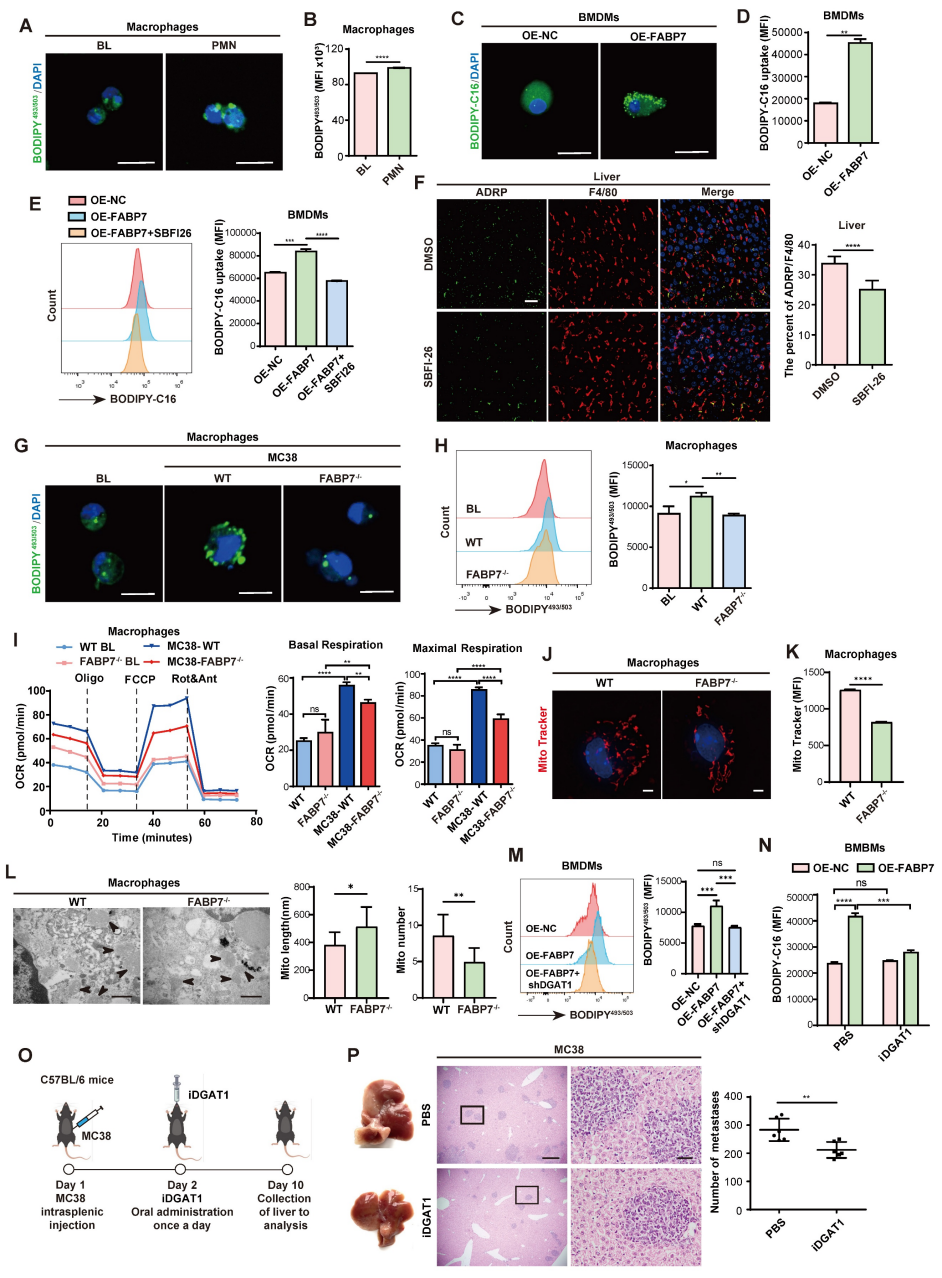
**FABP7 promotes LDs accumulation via mediating DGAT1 in PMN liver macrophages.** (A-B) Representative images of lipid staining (A) and flow cytometry analysis of lipid levels (B) of liver macrophages isolated from tumor-free mice (BL) and MC38-bearing mice in PMN (n=3). Scale bars, 10 µm. (C-D) Fatty acid uptake in OE-FABP7 BMDMs were analyzed by lipid staining (C) and flow cytometry (D, n=3). Scale bars, 10 µm. (E) The MFI of fatty acid uptake in OE-FABP7 BMDMs following SBFI-26 treatment (n=3). (F) IF staining of ADRP (green) and F4/80 (red) in liver sections from SBFI-26-treated MC38-bearing mice in PMN, with the ADRP/F4/80 ratio quantified (n=3). Scale bars, 20 µm. (G-H) Lipid content in liver macrophages isolated from normal liver, WT and FABP7^-/-^ mice bearing MC38 in PMN was measured by IF (G) and flow cytometry (H, n=3). Scale bars, 10 µm. (I) Oxygen consumption rate (OCR) of the liver macrophages isolated from WT and FABP7^-/-^ mice with or without MC38 tumors (n=3). (J-K) Representative images (J) and flow cytometry analysis (K) of MitoTracker Red staining of macrophages isolated from WT and FABP7^-/-^ mice bearing MC38 tumors in PMN (n=3). Scale bars, 3 µm. (L) Representative electron microscopy images and statistical analyses of length and number of mitochondrial in macrophages isolated from WT and FABP7^-/-^ mice bearing MC38 tumors in PMN (n=3). Scale bars, 500 nm. (M) Flow cytometry analysis of lipid content in OE-FABP7 BMDMs following shDGAT1 knockdown (n=3). (N) Flow cytometry analysis of lipid content in OE-FABP7 BMDMs with and without DGAT1 inhibitor treatment (n=3). (O) Schematic diagram of DGAT1 inhibitor administration in MC38 tumor-bearing mice. (P) Representative images of liver tissues (left) and HE staining of liver tissues (middle) from MC38 bearing mice treated with DGAT1 inhibitor, with metastatic foci counts quantified (n=6). Scale bars, 200 μm (P, left of the middle panel) or 20 μm (P, right of the middle panel). MFI: mean fluorescence intensity. Data are presented as the mean ± SEM. p values were determined by unpaired two-tailed Student's t-test (B, D, F, K-L and P), one-way ANOVA (E, H, I and M) or two-way ANOVA (N). * p < 0.05, ** p < 0.01, *** p < 0.001, **** p < 0.0001; ns, not significant.

Tumor microenvironment (TME) conditions trigger metabolic reprogramming in macrophages, subsequently shaping their functional phenotypes^39^, ^40^. Lipidomic analysis revealed markedly elevated long-chain fatty acids (LCFAs)—particularly long-chain polyunsaturated fatty acids (PUFAs)—in liver PMN tissues of MC38 tumor-bearing mice (Table S2). Consistent with this, liver macrophages isolated at the PMN stage from these mice showed substantially higher LD accumulation compared to macrophages from tumor-free mice (Figure 4A, 4B). As FABP7 facilitates fatty acid uptake and transport, we overexpressed it (OE-FABP7) in BMDMs. OE-FABP7 BMDMs exhibited enhanced LCFA uptake and increased LD accumulation versus control *in vitro* (Figure 4C, 4D; Figure S5A). This effect was abolished by the FABP7 inhibitor SBFI-26 (Figure 4E). *In vivo* validation demonstrated reduced adipophilin (ADRP; an essential LD structural protein) in liver macrophages from SBFI-26-treated mice (Figure 4F). Similarly, FABP7^-/-^ mice displayed diminished LD accumulation in liver PMN macrophages (Figure 4G and 4H). Notably, M2 polarization depends on FAO^41^. In IL-4/IL-10-polarized M2 macrophages—which showed elevated FABP7 expression (Figure S4A) and LCFA uptake exceeded those in M0 or LPS/IFN-γ-polarized M1 macrophages (Figure S5B, S5C).

To elucidate the mechanism role of FABP7 in macrophages, we performed mRNA sequencing on liver macrophages isolated from WT and FABP7^-/-^ mice with MC38-induced liver metastases. This revealed 1,529 DEGs, with PPAR signaling and fatty acid metabolism emerging as the top two enriched KEGG pathways (Figure S5D). However, qPCR analysis revealed no significant changes in PPAR target gene expression (PPARα, PPARβ, PPARγ, and PGC-1α) in FABP7 KO BMDMs (Figure S5E). Gene enrichment analysis further indicated that FABP7 KO impaired citrate cycle and oxidative phosphorylation pathways (OXPHOS) (Figure S5F). Mitochondrial stress testing confirmed reduced basal and maximal respiration in FABP7 KO macrophages from metastasized liver (Figure 4I), demonstrating impaired mitochondrial function. Subsequent morphological analysis via Mito Tracker staining and transmission electron microscopy (TEM) showed that FABP7 deletion decreased mitochondrial fission while increasing fusion in MMN-stage macrophages (Figure 4J-4L). Conversely, fission was elevated in PMN-stage macrophages and OE-FABP7 BMDMs (Figure S5G-S5L). To validate FAO dependence, we treated macrophages with the FAO inhibitor etomoxir (Eto). qPCR analysis showed that elevated M2-marker expression in OE-FABP7 BMDMs was abolished by etomoxir treatment (Figure S5M), indicating FABP7 potentiates mitochondrial oxidative metabolism. Given mitochondria primarily generate energy from glucose, glutamine, and fatty acids, we performed substrate dependency assays. FABP7 KO specifically reduced macrophage reliance on fatty acids in metastasized livers (Figure S5N), and palmitate (PA) oxidation stress tests confirmed diminished PA-driven mitochondrial respiration (Figure S5O). Isotope tracing experiments using ^13^C-labeled acetate and palmitate demonstrated reduced incorporation of these substrates into tricarboxylic acid (TCA) cycle intermediates (fumarate, oxaloacetate, and α-KG) in FABP7 KO BMDMs (Figure S6A-S6C). Similarly, ^13^C-PA-derived acetyl-CoA and TCA cycle metabolites (citrate, succinate, and α-KG) showed decreased enrichment in FABP7 KO BMDMs (Figure S6D-S6F). These findings collectively establish FABP7 as a key regulator of lipid metabolism in macrophage mitochondria.

LDs are dynamic organelles regulated by key lipid metabolism enzymes, including the LD biogenesis enzymes diacylglycerol acyltransferase 1 (DGAT1) and DGAT2 and degradation regulators adipose tissue triacylglycerol lipase (ATGL), hormone-sensitive lipase (HSL), and monoacylglycerol lipase (MGL)^42^, ^43^. FABP7 primarily induced DGAT1 upregulation (Figure S5P, S5Q). To investigate whether FABP7-mediated LD accumulation depends on DGAT1, we simultaneously overexpressed FABP7 and silenced DGAT1 in macrophages. Flow cytometry analysis revealed that while FABP7 OE markedly increased LD formation, DGAT1 knockdown abolished this effect (Figure 4M). Consistent with this, pharmacological DGAT1 inhibitor (iDGAT1, A922500) abrogated FABP7-induced lipid accumulation and M2 marker expression in BMDMs (Figure 4N; Figure S5R), and attenuated liver metastasis *in vivo* (Figure 4O, 4P). Together, these results demonstrate that FABP7 promotes LD accumulation in liver PMN macrophages by upregulating DGAT1, thereby enhancing macrophage FAO and promoting M2 polarization.

### Hypoxia-induced FABP7 in macrophages promotes LD accumulation in the liver PMN

**Figure 5:**
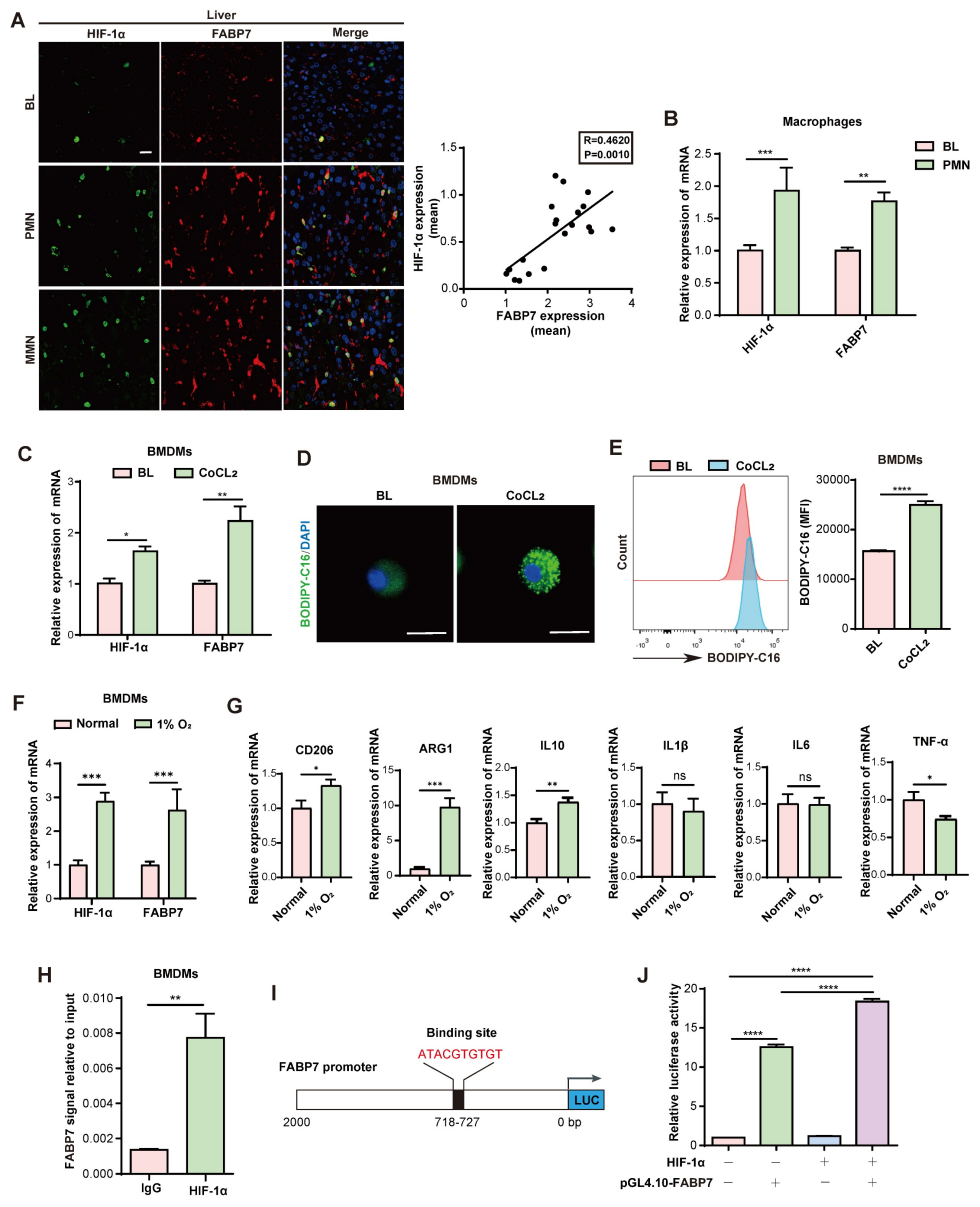
**Hypoxia mediates FABP7 expression and lipid accumulation in liver macrophages.** (A) Representative IF images of HIF-1α (green) and FABP7 (red) in livers from MC38 tumor-bearing mice in PMN or MMN, compared with normal liver tissue (n=3). The right panel displayed their expression correlation (Pearson R=0.4620, p=0.001). Scale bars, 20 µm. (B) qPCR analysis of HIF-1α and FABP7 expression level in macrophages isolated from MC38 tumor-bearing mice in PMN (n=3). (C) qPCR analysis of HIF-1α and FABP7 expression levels in CoCL_2_-treated BMDMs (n=3). (D-E) Fatty acid uptake in CoCL_2_-induced BMDMs analyzed by lipid staining (D) and flow cytometry (E, n=3). Scale bars, 10 µm. (F-G) qPCR analysis of HIF-1α, FABP7 and M1/M2 macrophage-relative gene expression in BMDMs cultured under hypoxic conditions (1% O_2_; n=3). (H) ChIP-qPCR demonstrated HIF-1α binding to FABP7 promoter regions (n=3). (I) Schematic diagrams depicted the luciferase reporter vector binding sites. (J) Dual luciferase assay in 293T cells co-transfected with pGL4.0-FABP7 and either HIF-1α or control vectors (n=3). CoCL_2_, cobalt dichloride. Data are presented as the mean ± SEM. p values were determined by Pearson correlation (A), unpaired two-tailed Student's t-test (E and G-H), one-way ANOVA (J) or two-way ANOVA (B-C and F). * p < 0.05, ** p < 0.01, *** p < 0.001, **** p < 0.0001; ns, not significant.

To investigate whether hypoxia induces FABP7 expression and drives LD accumulation in liver PMN macrophages, we performed histological analyses of MC38 tumor-bearing mice. These revealed strong positive correlations between FABP7 and HIF-1α expression in both PMN and MMN liver tissues (Figure 5A). This correlation was corroborated in CRC liver metastasis patients (GEO dataset GSE131418)^44^ (Figure S7A). While HIF-1α levels remained unchanged in lung and bone PMN (Figure S7B, S7C), liver macrophages from MC38 tumor-bearing mice exhibited significant upregulation of both HIF-1α and FABP7 compared to controls (Figure 5B). *In vitro*, cobalt dichloride (CoCL_2_)-induced hypoxia markedly increased HIF-1α and FABP7 mRNA in macrophages without altering HIF-1α in OE-FABP7 macrophages (Figure 5C; Figure S7D, S7E), concurrent with enhanced LD accumulation (Figure 5D, 5E; Figure S7F, S7G). Physiological hypoxia (1% O₂, 48 h)​​ similarly upregulated HIF-1α and FABP7 in macrophages (Figure 5F) and induced M2 polarization (Figure 5G). HIF-1α gain- and loss-of-function experiments in BMDMs demonstrated that HIF-1α overexpression upregulated FABP7 mRNA, while knockdown reduced it (Figure S7H, S7I). Chromatin immunoprecipitation (ChIP)-qPCR confirmed HIF-1α enrichment at the FABP7 promoter (Figure 5H). In silico analysis (TFtarget database) identified a high-confidence HIF-1α binding site (score: 0.86) within the 2-kb upstream region of the FABP7 transcription start site (Figure 5I). Luciferase reporter assays demonstrated that HIF-1α OE enhanced FABP7 promoter activity in 293T cells transfected with WT pGL4.10-FABP7 (Figure 5J). Collectively, these findings demonstrate that hypoxia-induced HIF-1α transcriptionally activates FABP7 to drive LD accumulation in liver PMN macrophages.

### FABP7-mediated lipid-laden macrophages metabolically reprograms CD8^+^ T cells, leading to their dysfunction

**Figure 6:**
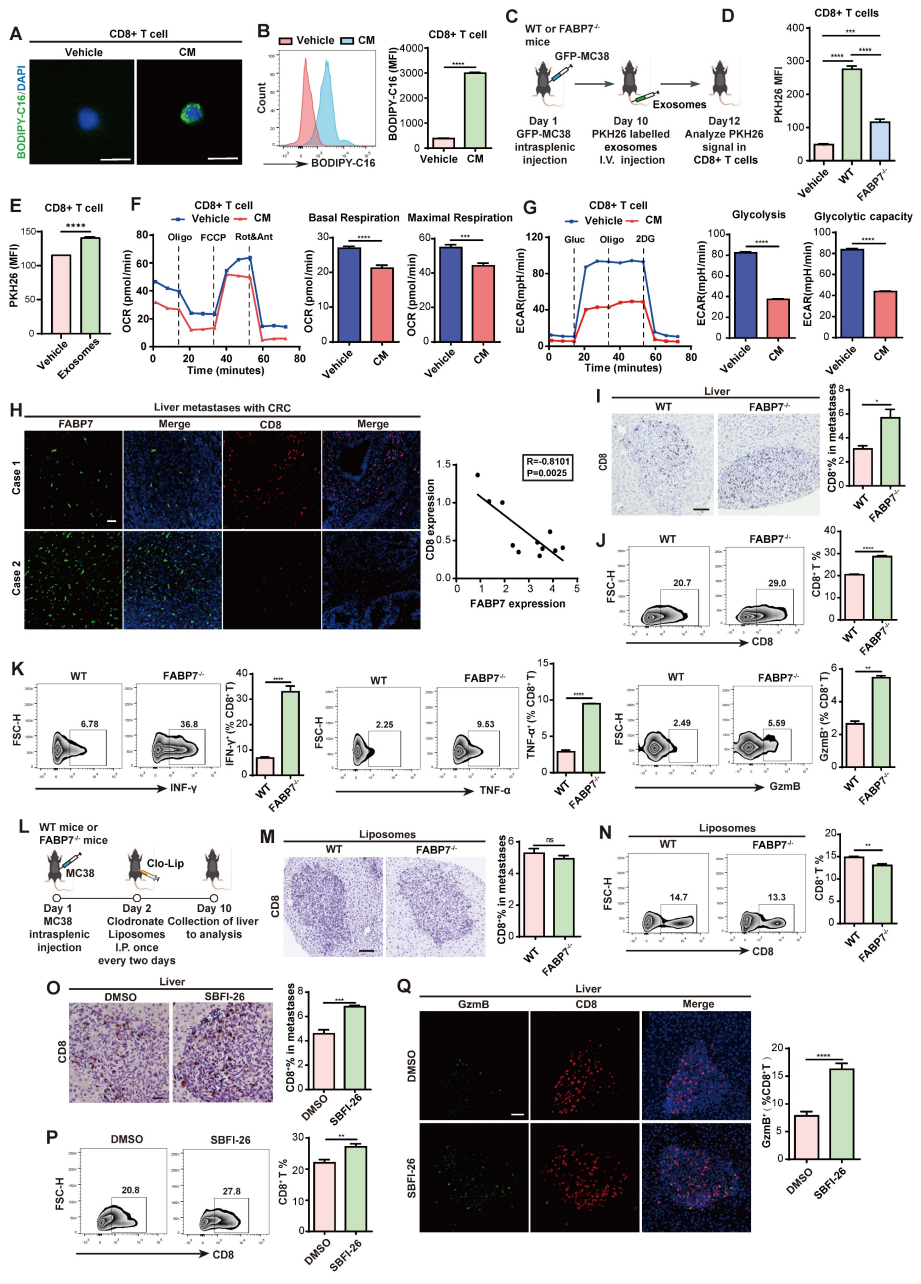
** FABP7 mediates lipid-laden macrophages metabolically reprograms CD8^+^ T cells leading to their dysfunction.** (A-B) Confocal microscopy (A) and flow cytometry (B) revealed lipids incorporation into CD8^+^ T cells following culture with BODIPY-C16 labeled BMDMs-CM (n=3). Scale bars, 10 µm. (C) Pattern diagram illustrated exosomes uptake by CD8^+^ T cells in murine models. (D) Flow cytometry analysis of exosomal content within CD8^+^ T cells from WT and FABP7^-/-^ mice with MC38 metastases (n=3). (E) Flow cytometry analysis of exosomal contents in CD8^+^ T cells incubated with BMDM-derived exosomes (n=3). (F-G) Real-time analysis of oxygen consumption rate (OCR) in CD8^+^ T cells exposed to BODIPY-C16 labeled BMDM-CM. Basal respiration ΔOCR and maximal respiration ΔOCR were quantified (n=3). (H) Representative IF images of FABP7 (green) and TIL-CD8^+^ T cell (red) in liver tissues from CRC patients with metastases. The right panel displayed the inverse correlation between CD8^+^ T cell density and FABP7 expression (Pearson R = -0.8101, p = 0.0025; n = 11). Scale bars, 50 µm. (I) IHC staining of CD8^+^ T cell subpopulation in liver metastatic niches of WT and FABP7^-/-^ mice with MC38 metastasis (n=5). Scale bars, 20 µm. (J-K) Flow cytometry analysis the TIL-CD8^+^ T cell numbers (J) and their IFN-γ, TNF-a, and GzmB expression (K) in livers of MC38 tumor-bearing WT and FABP7^-/-^ mice (n=3). (L) Pattern diagram of MC38 tumor-bearing WT and FABP7^-/-^ mice treated with clodronate liposomes. (M-N) IHC staining (M) and flow cytometry analysis (N) of TIL-CD8^+^ T cell in livers of clodronate-treated, MC38 tumor-bearing WT and FABP7^-/-^ mice (n=3). Scale bars, 20 µm (M). (O-P) IHC staining (O) and flow cytometry analysis (P) of TIL-CD8^+^ T cells in livers of MC38 tumor-bearing C57BL/6 mice treated with SBFI-26 (n=4). Scale bars, 20 µm. (Q) IF staining of GzmB expression in TIL-CD8^+^ T cells in livers of MC38 tumor-bearing C57BL/6 mice treated with SBFI-26, with GzmB^+^ percentages quantified for CD8^+^ T cell populations (n=4). Scale bars, 20 µm. CM, conditioned medium; TIL, tumor infiltrating lymphocytes. Data are presented as mean ± SEM. p values were determined by unpaired two-tailed Student's t-test (B, E-G, I-K and M-Q), one-way ANOVA (D) or Pearson correlation (H). * p < 0.05, ** p < 0.01, *** p < 0.001, **** p < 0.0001; ns, not significant.

Lipid metabolism orchestrates tumor-microenvironment crosstalk^22^. Given the pivotal role of CD8^+^ T cells in antitumor immunity^45^, ^46^, we investigated how FABP7-mediated lipid accumulation in macrophages affects their function. We pre-incubated BMDMs with fluorescently labeled LCFA (BODIPY-C16) to generate lipid-laden macrophages, then collected conditioned medium (CM). Strikingly, CD8⁺ T cells cultured in this CM internalized fluorescent lipids, forming LD-like structures (Figure 6A, 6B), indicating intercellular lipid transfer from. Since extracellular vesicles (EVs) mediate lipid trafficking and cellular communication^47^-^49^, we fractionated CM using a 100-kDa filter to separate free fatty acids (<100 kDa) from vesicle-bound lipids (>100 kDa) (Figure S8A)^22^. The >100-kDa fraction induced significantly higher lipid uptake in CD8^+^ T cells (Figure S8B), suggesting exosomes-mediated transfer. This was confirmed by PKH26 labeling: flow cytometry showed enhanced PKH26 signal in CD8^+^ T cells exposed to CM from labeled BMDMs (Figure S8C). Nanoparticle tracking and western blot (CD63/TSG101) verified exosome enrichment after ultracentrifugation (Figure S8D, S8E). *In vivo*, CD8^+^ T cells internalized lipid-laded BMDM-derived exosomes in an FABP7-dependent manner (Figure 6C, 6D); this uptake was replicated *in vitro* (Figure 6E). CM-exposed CD8^+^ T cells exhibited reduced oxidative phosphorylation and glycolysis (Figure 6F, 6G), with mitochondrial fuel assays showing diminished glucose dependence and attenuated PA-driven respiration (Figure S8F, S8G). Concurrently, exhaustion markers (PD1⁺, TIM3⁺) increased (Figure S8H, S8I), while effector molecules (IFN-γ, TNF-α, GzmB) were downregulated (Figure S8J). These findings demonstrate that lipid-laden macrophages deliver lipids via exosomes to metabolically reprogram CD8^+^ T cells, impairing their antitumor function.

We then assessed how FABP7 influences CD8^+^ T cell activity. Clinical analysis revealed an inverse correlation between FABP7 expression and tumor-infiltrating CD8^+^ T cell (TIL) density in liver metastases from CRC patients (Figure 6H). Consistently, FABP7^-/-^ mice exhibited increased CD8^+^ TILs compared to WT control during MC38-induced liver metastasis (Figure 6I, 6J). These T cells showed enhanced functionality—evidenced by elevated IFN-γ, TNF-α, and GzmB expression alongside reduced PD1 and TIM3 exhaustion markers (Figure 6K; Figure S8K). Antigen-specific validation using OVA-MC38 models confirmed increased tetramer^+^ CD8^+^ T cells with heightened TNF-α and GzmB production in FABP7^-/-^ mice (Figure S8L). Notably, clodronate-mediated macrophage ablation abolished these T cell enhancements (Figure 6L-6N; Figure S9A, S9B). Clodronate treatment effectively eliminated liver macrophages while sparing lung macrophages and causing only partial depletion in spleen and bone (Figure S9C). Sequential depletion experiments revealed that FABP7 knockout's antimetastatic effect required both macrophages and CD8+ T cells, as individual depletion of either population exacerbated metastases, with combined depletion showing additive effects (Figure S9D-S9F). Pharmacological FABP7 inhibition with SBFI-26 mirrored genetic ablation, increasing functional CD8^+^ TILs and boosting IFN-γ, TNF-α, and GzmB production (Figure 6O-6Q; Figure S9G). These findings suggest FABP7 deletion in liver macrophages potentiates CD8^+^ T cell-mediated antitumor immunity.

### FABP7 mediates lipid-laden macrophages to deliver LDs to support metastatic colonization

**Figure 7:**
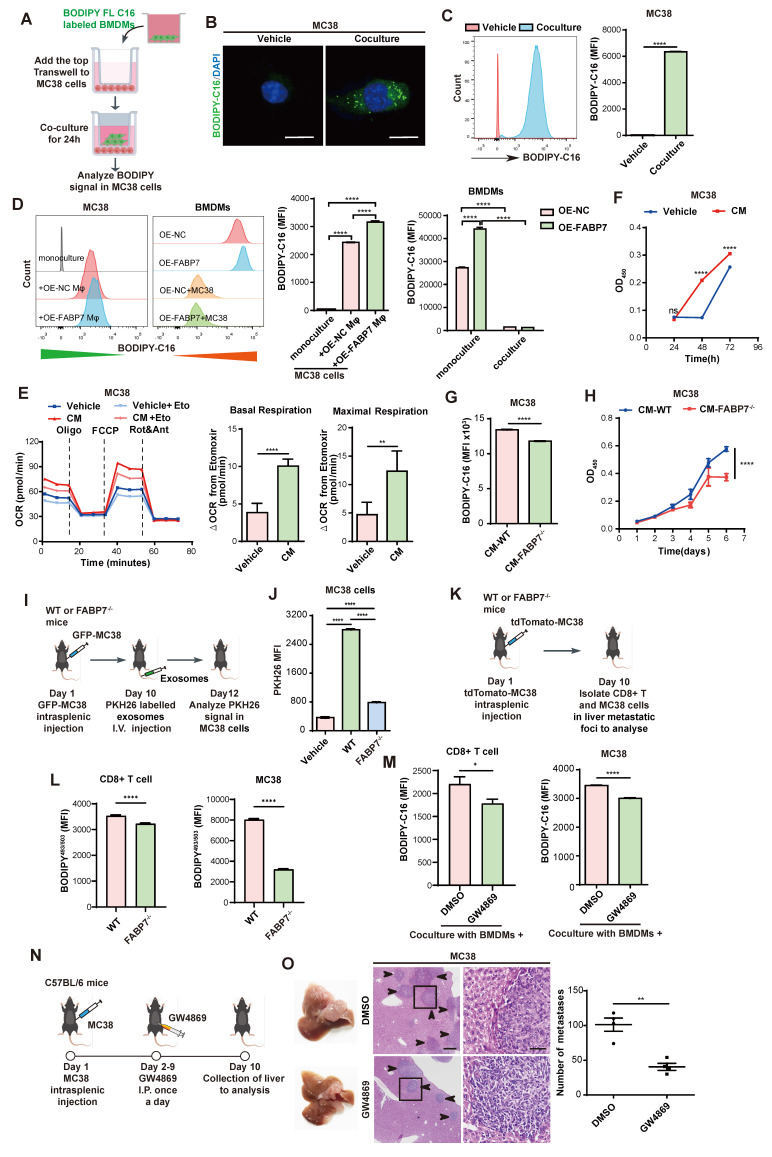
**FABP7 mediates lipid-laden macrophages transport lipids to support metastatic colonization.** (A-C) As depicted in the schematic (A), the lipids incorporated into MC38 cells were detected with confocal microscopy (B) and flow cytometry (C) after coculture with BODIPY-C16 labeled BMDMs (n=3). Scale bars, 10 µm. (D) The MFI of lipids in MC38 cells cocultured with BMDMs preloaded with BODIPY-C16 determined by flow cytometry (n=3). (E) Real-time oxygen consumption rate (OCR) analysis of MC38 cells treated with CM from BODIPY-C16 labeled BMDMs and the FAO inhibitor etomoxir. Basal respiration ΔOCR and maximal respiration ΔOCR were quantified (n=3). (F) The proliferation of MC38 cells was measured after incubated with CM from BODIPY-C16 labeled BMDMs via CCK8 assay (n=5). (G) Flow cytometry analysis of lipid contents in MC38 cells cultured with CM from BODIPY-C16 labeled KO-FABP7 or control BMDMs (n=3). (H) CCK8 assays measured MC38 cells proliferation following incubation with CM from BODIPY-C16 labeled KO-FABP7 or control BMDMs (n=5). (I) Schematic image illustrates exosome uptake by tumor cells in mice. (J) Flow cytometry analysis of exosomes content in tumor cells from livers of WT and FABP7^-/-^ mice with MC38 metastases (n=3). (K-L) The schematic (K) illustrated flow cytometric analysis of LD contents in CD8^+^ T and MC38 cells isolated from liver metastases of MC38-bearing WT and FABP7^-/-^ mice (L, n=3). (M) Flow cytometry analysis of lipid contents in CD8^+^ T and MC38 cells cocultured with BODIPY-C16 labeled BMDMs treated with inhibitor GW4869 (n=3). (N) Pattern diagram of using inhibitor GW4869 to treat MC38 bearing mice. (O) Representative images of liver tissues (left) and HE staining of liver tissues (middle) from MC38 bearing mice treated with GW4869. The number of liver metastatic colonization was shown on the right (n=4). Scale bars, 200 μm (O, left of the middle panel) or 20 μm (O, right of the middle panel). Data are presented as mean ± SEM. p values were determined by unpaired two-tailed Student's t-test (C, E-H and L-M) or one-way ANOVA (D and J). * p < 0.05, ** p < 0.01, *** p < 0.001, **** p < 0.0001; ns, not significant.

We next examined whether macrophages transfer stored lipids to tumor cells. MC38 cells were cocultured with BMDMs pre-incubated with BODIPY-C16 (Figure 7A). IF and flow cytometry revealed increased fluorescence in MC38 cells but decreased signals in BMDMs after co-culture, with OE-FABP7 BMDMs exhibiting enhanced lipid transfer efficiently (Figure 7B-7D). CM from labeled BMDMs similarly delivered lipids to MC38 cells (Figure S10A, S10B), which localized to tumor cell mitochondria (Figure S10C). This lipid transfer induced metabolic reprogramming in MC38 cells, evidenced by upregulated FAO pathway genes (Figure S10D) and increased FAO phosphorylation (Figure 7E). Mitochondrial fuel testing demonstrated greater fatty acid dependence and elevated maximum PA respiration in CM-treated MC38 cells (Figure S10E, S10F). Corresponding to enhanced lipid utilization, MC38 cells displayed accelerated proliferation (Figure 7F). Conversely, FABP7 KO attenuated lipid transfer and diminished tumor cell proliferation (Figure 7G, 7H), while reducing tumor cell death upon coculture (Figure S10G). These findings demonstrate that macrophage-derived lipids promote tumor cell proliferation through metabolic reprogramming.

To determine whether macrophage-derived lipids transfer to tumor cells via exosomes, we fractionated macrophage-CM by molecular weight. The >100 kDa fraction induced significantly stronger fluorescent signals in MC38 cells compared to the <100 kDa fraction (Figure S10H-S10J), suggesting exosome-dependent lipid transfer. Flow cytometry confirmed enhanced PKH26 signal in MC38 cells exposed to CM from labeled BMDMs (Figure S10K), with direct visualization confirming exosomal uptake by MC38 cells (Figure S10L, S10M). Crucially, *in vivo* tracking revealed FABP7-dependent uptake of lipid-laden BMDM-derived exosomes by MC38 cells (Figure 7I, 7J). Consistently, CD8^+^ T cells and MC38 cells from FABP7^-/-^ liver metastatic foci exhibited reduced LD content (Figure 7K, 7L). Lipidomic analysis of liver PMN macrophage CM revealed reduced triglyceride (TG) but unchanged free fatty acid (FFA) levels in FABP7^-/-^ samples (Figure S10N), with purified exosomes showing identical selective TG deficiency (Figure S10O). Pharmacological inhibition of exosome secretion (GW4869) blocked lipid transfer to both CD8^+^ T cells and MC38 cells (Figure 7M), suppressed tumor proliferation, and reduced hepatic metastatic burden *in vivo* (Figure 7N, 7O; Figure S10P). These findings establish that FABP7 enables macrophages to selectively deliver triglycerides via exosomes, reprogramming tumor metabolism to drive metastatic growth.

## Discussion

Metastasis remains an urgent challenge in current cancer therapies. Lipid metabolic reprogramming is increasingly recognized as a key driver of tumor progression^50^. Lung fibroblasts enhance tumor cell proliferation and metastasis by secreting cathepsin B (CTSB), which upregulates stearoyl CoA desaturase 1 (SCD1) expression and subsequently increases fatty acid content in tumor cells^51^. Although lipid metabolic reprogramming drives PMN formation^22^, the molecular mechanisms linking macrophage-mediated lipid dynamics to hepatic PMN establishment remain poorly defined. Our study uncovered a ​​FABP7-centered lipid cascade​​ that links macrophage metabolic reprogramming to immunosuppressive niche formation. Mechanistically, hypoxia-induced HIF-1α transcriptionally activates FABP7, driving ​​DGAT1-dependent LD biogenesis in macrophages. This coordinated process potentiates M2 polarization and facilitates exosomal lipid shuttling to both CD8^+^ T cells and tumor cells, ultimately driving metastatic progression.

FABP7 is a member of the fatty acid-binding protein family that facilitates cellular fatty acid uptake, transport, and metabolic regulation while influencing gene expression and growth pathways^52^. Its established role in tumor development^53^ contrasts with previously unexplored functions within the TME. We identified selective FABP7 upregulation in liver macrophages during PMN formation - a pattern absented in lung, bone, or spleen macrophages. Hepatic macrophages comprise two ontogenetically distinct populations: embryo-derived Kupffer cells (KCs) and bone marrow-derived monocyte-derived macrophages (MoMFs) 54. FABP7 upregulation occurred predominantly in KCs during PMN formation, extending to both KCs and MoMFs in established metastatic niches. This expression pattern was mechanistically driven by the organ's hypoxic microenvironment - a known catalyst for hepatic tumor progression^55^, wherein tumor-induced ​​HIF-1α​​ activation directly bound the FABP7 promoter. The ​​hypoxia-HIF-1α-FABP7 axis​​ accounted for FABP7's selectively induction in liver-resident KCs during PMN initiation and subsequent upregulation in recruited MoMFs during overt metastasis. Importantly, HIF-1α depletion eliminated FABP7 induction in tumor-conditioned macrophages, confirming its upstream regulatory role.

Patients with liver metastases exhibit poor prognosis and diminished treatment response^56^. Existing therapies show limited efficacy, posing significant challenges in managing metastatic progression^57^. We observed progressive upregulation of FABP7 in macrophages during liver PMN formation and metastatic colonization in CRC and PDAC patients, correlating strongly with adverse clinical outcomes. To explore FABP7's therapeutic potential, we generated global FABP7 knockout mice. To evaluate systemic knockout effects across cell types, we found FABP7 expression restricted to F4/80^+^ macrophages within the liver PMN, with undetectable expression in neutrophils, lymphocytes, or fibroblasts. Systemic KO did not impair macrophage functions in lungs, bones, or spleens nor alter Treg activity. Notably, both genetic ablation and pharmacological inhibition with SBFI-26 consistently suppressed metastasis, confirming FABP7 as a promising druggable target specific to the liver microenvironment.

Macrophages exhibit functional plasticity, polarizing toward pro-inflammatory (M1) or pro-tumorigenic (M2)^58^. We demonstrated that FABP7 triggered M2 polarization in liver PMNs. Crucially, both genetic FABP7 ablation and pharmacological inhibition (SBFI-26—previously used in murine inflammatory pain models^36^) suppressed M2 polarization. Given that lipid accumulation confers metastasis-promoting capacities in immune cells during PMN and MMN formation^48^, ^59^, and M2 activation requires FAO, we investigated FABP7's metabolic role. Tumor-bearing mice exhibited elevated LCFAs in liver PMNs. FABP7 enhanced fatty acid uptake and LD accumulation in macrophages *in vivo* and *in vitro*, concomitant with heightened mitochondrial respiration. Substrate dependency assays revealed increased fatty acid utilization in FABP7-expressing macrophages. Isotopic tracing with ^13^C-acetate and ^13^C-PA in FABP7-KO versus WT BMDMs demonstrated suppressed lipid flux into TCA cycle intermediates—confirming FABP7 's essential role in macrophage lipid utilization. Mechanistically, FABP7 channeled fatty acid to DGAT1, the terminal triglyceride synthesis enzyme, triggering extensive LD biogenesis. DGAT1 knockdown reversed both LD accumulation and M2 polarization in OE-FABP7 macrophages. The resulting LD accumulation sustained M2 commitment via FAO-dependent mitochondrial respiration, creating a feedforward loop where lipid storage begets protumor phenotypes.

Metabolic crosstalk within the TME critically regulates immune functionality^60^. We found that lipid-mediated communication between macrophages and CD8^+^ T cells orchestrated the immunosuppressive landscape of liver metastasis. Additionally, our data demonstrated that CD8^+^ T cells internalized lipids from BMDM-derived exosomes. This LD burden disrupts lipid metabolism and impairs cytotoxic function in T cells^61^, as evidenced by diminished mitochondrial respiration and glycolytic capacity following macrophage-derived lipid transfer. These cells simultaneously showed elevated expression of exhaustion markers (PD1⁺, TIM3⁺) and reduced production of effector molecules (IFN-γ, TNF-α, GzmB). Importantly, FABP7 deletion significantly decreased exosomal triglyceride content, thereby limiting lipid droplet accumulation in CD8^+^ T cells. In MC38-induced liver metastasis models, FABP7^-/-^ mice harbored more CD8^+^ TILs than wild-type controls. These T cells displayed improved functionality, characterized by higher IFN-γ, TNF-α, and GzmB levels alongside lower PD1 and TIM3 expression. Pharmacological inhibition of FABP7 using SBFI-26 recapitulated the genetic knockout phenotype, enhancing both the quantity and effector function of CD8^+^ TILs. These findings suggest FABP7 deletion in liver macrophages enhances CD8^+^ T cell-mediated antitumor immunity by preventing lipid-induced metabolic dysfunction.

We hypothesized that the LDs released by lipid-laden macrophages could be internalized by metastatic cancer cells. Our findings demonstrated that FABP7-mediated lipid transfer from macrophages to tumor cells increased their dependence on mitochondrial fatty acid oxidation, thereby facilitating metastatic colonization. Extracellular vesicles (EVs) serve as essential mediators of intercellular lipid trafficking^48^. While previous studies describe tumor-to-macrophage lipid transfer via vesicles^62^ and tumor-derived exosomes rewiring dendritic cell metabolism^63^, our findings demonstrated a reciprocal mechanism whereby TG-rich exosomes​​ mediated lipid transfer from macrophages to both CD8^+^ T cells and metastatic cells. Pharmacological inhibition of exosome secretion using GW4869 effectively suppressed metastatic progression, confirming the essential role of vesicular transport.

In summary, our study demonstrates that hypoxia-induced FABP7 expression in macrophages drives PMN formation and hepatic metastasis by promoting LD accumulation and impairing CD8^+^ T cell function, ultimately supporting metastatic growth through metabolic reprogramming. FABP7^+^ macrophages transferred TG-enriched exosomes to CD8^+^ T cells, driving PD-1 upregulation, this interaction suggests therapeutic synergy between FABP7 inhibition and anti-PD1 immunotherapy in metastatic disease. These results establish FABP7 as a promising therapeutic target for intercepting liver metastasis.

## Supplementary Material

Supplementary figures and tables.

## Abbreviations

PMN: pre-metastatic niche
MMN: macro-metastatic niche
FABP7: fatty acid binding protein 7
CRC: colorectal cancer
PDAC: pancreatic ductal adenocarcinoma
TME: tumor microenvironment
MIF: migration inhibitory factor
KCs: Kupffer cells
OS: overall survival
RFS: relapse-free survival
PUFAs: polyunsaturated fatty acids
DHA: docosahexaenoic acid
FAO: fatty acid oxidation
BMDMs: bone marrow-derived macrophages
qPCR: Quantitative real-time PCR
IHC: immunohistochemical
IF: immunofluorescence
LD: lipid droplet
HIF-1α: hypoxia inducible factor-1 alpha
DGAT1: diacylglycerol acyltransferase 1
DGAT2: diacylglycerol acyltransferase 2
ATGL: adipose tissue triacylglycerol lipase
HSL: hormone-sensitive lipase
MGL: monoacylglycerol lipase
OE: overexpression
KO: knockout
SBFI-26: a-Truxillic acid 1-naphthyl ester
I.P.: intraperitoneally
ADRP: adipophilin
CoCL_2_: cobalt dichloride
CM: conditioned medium
