# Supplementary material for: FABP7-mediated lipid-laden macrophages drive the formation of pre-metastatic niche and liver metastasis
Source: Int J Biol Sci. 2025 Jun 23;21(10):4388–409. doi: 10.7150/ijbs.110750 (PMC12320231; doi:10.7150/ijbs.110750)
Supplement: Supplementary file 1 — Supplementary figures and tables. [file ijbsv21p4388s1.pdf]

Supplemental Figures

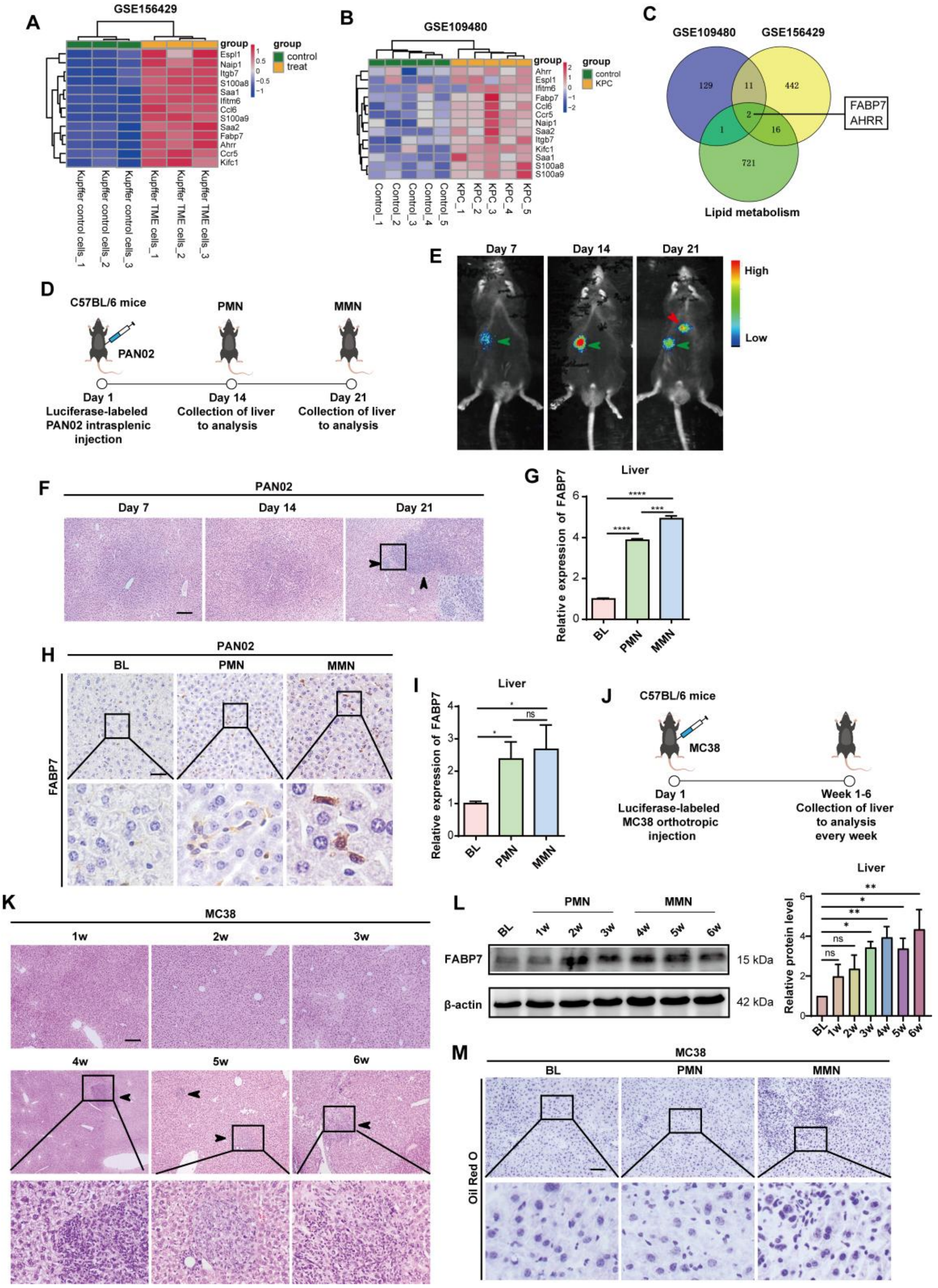

Figure S1. FABP7 is upregulated in livers at the PMN stage.

(A–B) Heat map showing representative differentially expressed genes identified in GSE156429 and GSE109480 datasets of liver from Gene Expression Omnibus (GEO) database. (C) Venn diagram of the number of co- and differentially expressed genes

among lipid metabolism and the two datasets. (D) Brief procedure displaying the PAN02 cells intrasplenic injection model for the liver metastasis process study. Green arrow indicated the splenic tumor in situ, while the red arrow indicated the liver metastasis. (E) Representative images of bioluminescence imaging of C57BL/6 mice intrasplenic injection luciferase-expressing PAN02 cells. (F) HE staining of liver tissue from mice after intrasplenic injection PAN02 cells. Representative images from day 7, day 14, and day 21 are shown. Scale bars, 100 μm. (G) qPCR analysis of mRNA levels of FABP7 expression in livers from mice without tumor (BL) and mice bearing MC38 in PMN and MMN (n=3). (H–I) IHC staining (H), and qPCR analysis of mRNA levels (I) of FABP7 expression in livers from mice without tumor (BL) and mice bearing PAN02 in PMN and MMN (n=3). Scale bars, 50 μm (H). (J) Brief procedure displaying the luciferase-expressing MC38 cells orthotopic transplantation liver metastasis model. (K) HE staining of liver tissue from mice after orthotopic transplantation MC38 cells. Representative images from week 1 to week 6 are shown. Scale bars, 100 μm. (L) Western blot analysis of protein levels of FABP7 expression in livers from mice at different stages (n=3). β-actin served as loading control. (M) Representative images of Oil Red O staining of liver from mice bearing MC38 in PMN and MMN. Scale bars, 100 μm. Data are presented as the mean ± SEM. p values were determined by one-way ANOVA (G, I and L): * $p < 0.05$, ** $p < 0.01$, *** $p < 0.001$, **** $p < 0.0001$; ns, not significant.

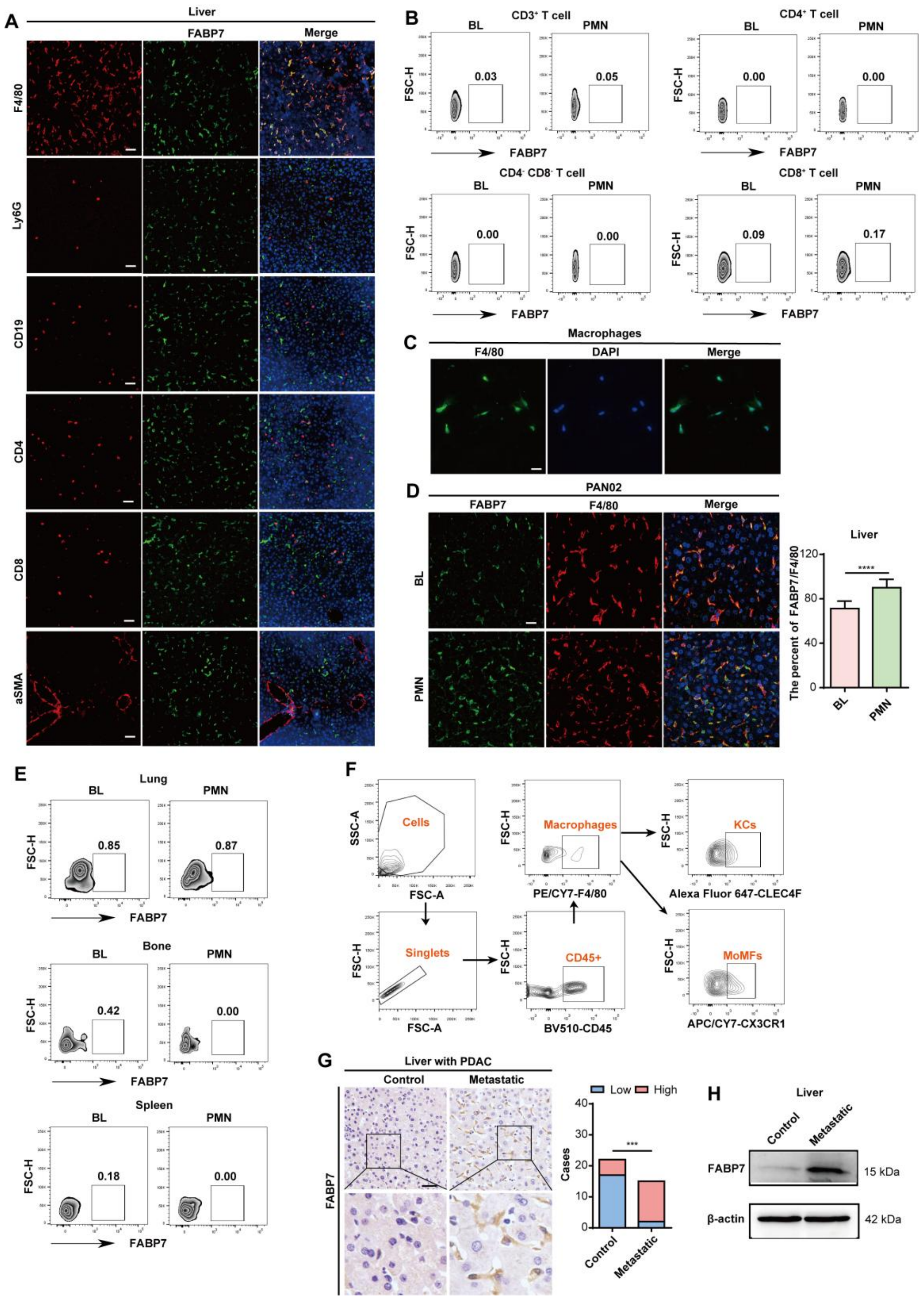

Figure S2. FABP7 is predominantly expressed in liver macrophages at PMN.

(A) Representative images of IF staining of FABP7 (green) expression in several cell types in the liver PMN of mice. Scale bars, 50 μm. (B) Flow cytometry analysis the number of FABP7 expression in different population of T cells (n=3). (C)

Representative IF image of F4/80 expression (green) in macrophages isolated from mice liver. Scale bars, 20 μm. (D) Representative images of IF staining of FABP7 (green) and F4/80 (red) in livers from mice without tumor (BL) and mice bearing PAN02 in PMN. The percentage of FABP7 in F4/80$^+$ macrophages was shown on the right (n=3). Scale bars, 20 μm. (E) Flow cytometry analysis of FABP7 expression in macrophages of PMN lung, bone and spleen (n=3). (F) Representative flow cytometry plot showing gating strategy used to analysis the number and FABP7 expression in different population of macrophages. (G) Representative images (original and magnified) of IHC staining of FABP7 expression in liver samples from PDAC patients with metastatic (n=15) and control livers (n=20). Statistical analysis using chi-square test was shown on the right. Scale bar, 20 μm. (H) Western blot analysis of FABP7 expression levels in liver samples from metastatic PDAC patients compared with controls (n=3). β-actin served as loading control. Data are presented as the mean ± SEM. p values were determined by unpaired two-tailed Student's t test (D). *** $p < 0.001$, **** $p < 0.0001$.

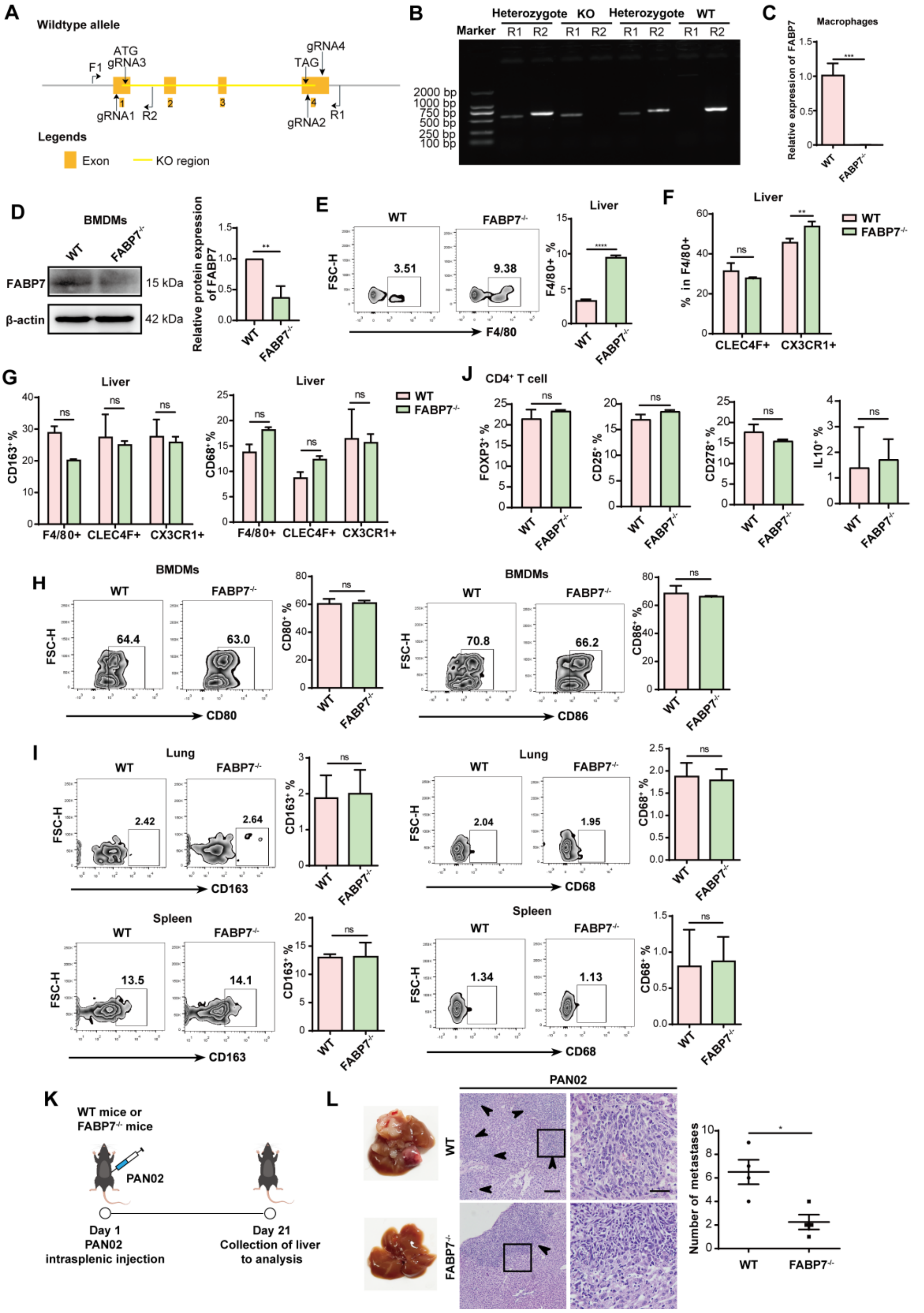

Figure S3. Generation of FABP7$^{-/-}$ mice.

(A) Schematic of the targeting strategy used to generate FABP7 knockout (KO, FABP7$^{-/-}$) mice. (B) The DNA levels of FABP7 detected in the WT and FABP7$^{-/-}$ mice. For the KO mice, there was one band with 538 bp; the heterozygote was two bands

with 538 bp and 614 bp, and the WT mice was one band with 614 bp. (C) qPCR analysis of mRNA levels of FABP7 in the liver macrophages isolated from normal WT and FABP7$^{-/-}$ mice (n=3). (D) Western blot analysis of protein levels of FABP7 in BMDMs from normal WT and FABP7$^{-/-}$ mice (n=3). β-actin served as loading control. (E–G) Flow cytometry analyses of the number (E and F) and M1, M2 macrophages (G) of total macrophages (F4/80^{+}), KCs (CLEC4F^{+}) or MoMFs (CX3CR1^{+}) in livers of normal WT and FABP7$^{-/-}$ mice (n=3 per condition). (H) Flow cytometry analyses of the CD80 and CD86 expression in BMDMs from normal WT and FABP7$^{-/-}$ mice (n=3). (I) Flow cytometry analyses of the M1 and M2 phenotype of macrophages in lung and spleen in WT and FABP7$^{-/-}$ mice at steady state (n=3 per condition). (J) Flow cytometry analyses of FOXP3^{+}, CD25^{+}, CD278^{+} and IL10^{+} in CD4^{+} T cells in livers of WT and FABP7$^{-/-}$ mice bearing with MC38 (n=3). (K–L) As depicted in the schematic (K), liver metastatic colonization was compared between WT and FABP7$^{-/-}$ mice bearing PAN02 cells (L, n=4). Scale bars, 200 μm (L, left of the middle panel) or 20 μm (L, right of the middle panel). Data are presented as the mean ± SEM. p values were determined by unpaired two-tailed Student's t-test (C, D, E, H–J and L) or two-way ANOVA (F–G). * $p < 0.05$, ** $p < 0.01$, *** $p < 0.001$; ns, not significant.

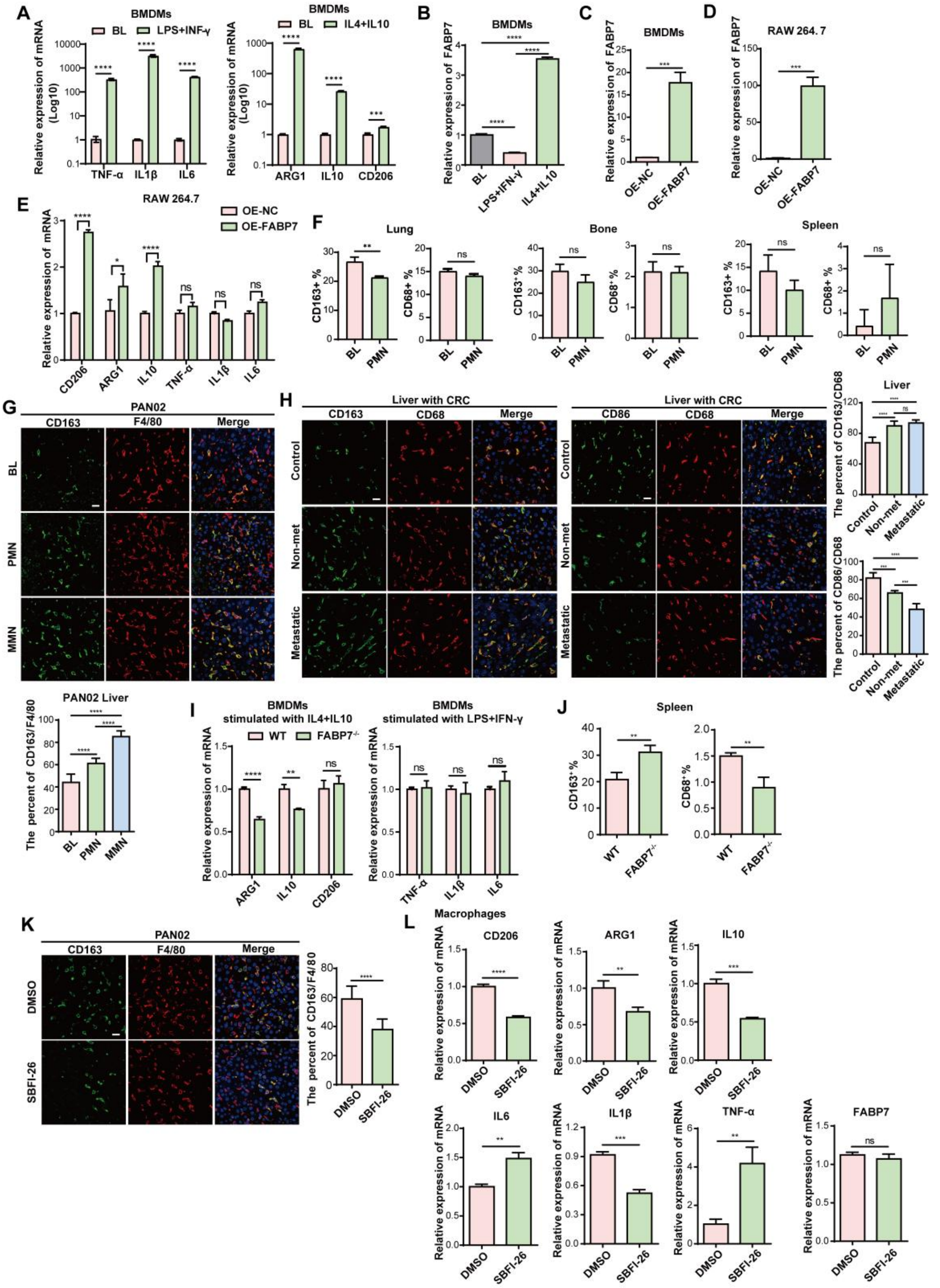

Figure S4. FABP7 increases macrophage M2 polarization.

(A–B) qPCR analysis of M1, M2 markers and FABP7 expression in BMDMs treated with LPS + IFN-γ or IL4 + IL10 (n=3). (C–D) qPCR analysis of FABP7 expression in OE-FABP7 BMDMs or RAW264.7 cells (n=3). (E) qPCR analysis of M2 and M1 markers expression in OE-FABP7 RAW264.7 cells compared to the control cells

(n=3). (F) Flow cytometry analysis of M2 and M1 markers expression in macrophages of PMN lung, bone and spleen (n=3). (G) Representative images of IF staining of CD163 (green) and F4/80 (red) in livers from mice bearing with PAN02 cells in PMN and MMN. The percentage of CD163 to F4/80 was shown on the bottom (n=3). Scale bars, 20 μm. (H) IF staining of CD163 (green) and CD86 (green) in CD68 (red) of liver tissues from CRC patients with or without metastatic and normal liver. The percentage of CD163 to CD68 was shown on the right (n=5 per condition). Scale bars, 20 μm. (I) qPCR analysis of M2 and M1 relative genes expression in WT and $FABP7^{-/-}$ BMDMs treated with IL4 + IL10 or LPS + IFN-γ (n=3). (J) Flow cytometry analyses of the M1 and M2 phenotype of macrophages in spleens from WT and $FABP7^{-/-}$ mice bearing with MC38 (n=3). (K) IF staining of CD163 (green) and F4/80 (red) in livers from C57BL/6 mice bearing PAN02 cells treated with DMSO or SBFI-26 in PMN. The percentage of CD163 to F4/80 was shown on the right (n=3). Scale bars, 20 μm. (L) qPCR analysis of M2, M1 relative genes and FABP7 expression in liver macrophages isolated from mice bearing MC38 cells treated with DMSO or SBFI-26 in PMN (n=3). Data are presented as the mean ± SEM. p values were determined by one-way ANOVA (B, G and H), two-way ANOVA (A, E and I) or unpaired two-tailed Student's t-test (C–D, F and J–L). * $p < 0.05$, ** $p < 0.01$, *** $p < 0.001$, **** $p < 0.0001$; ns, not significant.

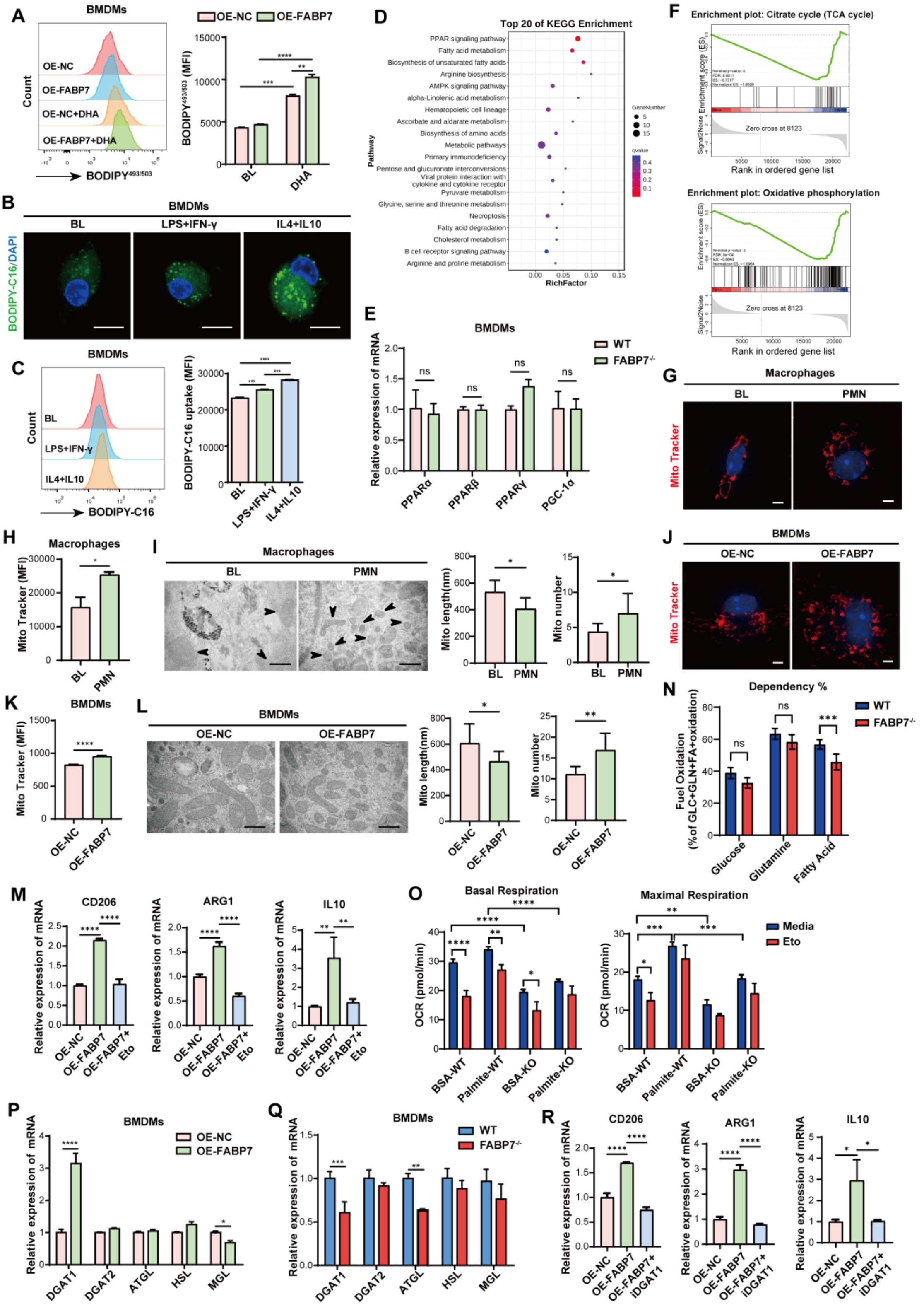

Figure S5. The affection of FABP7 in lipid metabolism in macrophages.

(A) Flow cytometry analysis of lipid levels in OE-FABP7 BMDMs cultured with or without DHA (n=3). (B–C) Lipid staining (B) and lipid levels determined by flow cytometry (C) in BMDMs treated with LPS+IFN-γ or IL4+IL10 (n=3). Scale bars, 10

μm (B). (D) Top 20 signaling pathway of KEGG pathway enrichment analysis of DEGs identified in macrophages isolated from WT and $FABP7^{-/-}$ mice bearing MC38 in MMN. (E) qPCR analysis of PPAR target gene (PPARα, PPARβ, PPARγ and PGC-1α) expressions in WT and $FABP7^{-/-}$ BMDMs (n=3). (F) GSEA enrichment of DEGs identified in macrophages isolated from WT and $FABP7^{-/-}$ mice bearing MC38 in MMN. (G–H) Representative images (G) and flow cytometry analysis (H) of MitoTracker Red staining of macrophages isolated from mice bearing MC38 in PMN (n=3). Scale bars, 3 μm. (I) Representative electron microscopy images and statistical analyses of length and number of mitochondrial in macrophages isolated from mice bearing MC38 in PMN (n=3). Scale bars, 500 nm. (J–K) Representative images (J) and flow cytometry analysis (K) of MitoTracker Red staining of OE-FABP7 BMDMs and control cells (n=3). Scale bars, 3 μm. (L) Representative electron microscopy images and statistical analyses of length and number of mitochondrial in OE-FABP7 BMDMs and control cells (n=3). Scale bars, 500 nm. (M) qPCR analysis of M2 relative genes in OE-FABP7 BMDMs and using the FAO inhibitor etomoxir (Eto) (n=3). (N) Fuel dependency of macrophages isolated from WT and $FABP7^{-/-}$ mice bearing MC38 to oxidize glucose, glutamine, and fatty acids of mitochondrial respiration (n=3). (O) Oxygen consumption rate (OCR) of palmitate in macrophages isolated from WT and $FABP7^{-/-}$ mice bearing MC38 (n=3). (P–Q) qPCR analysis of mRNA levels of LDs related enzyme genes in OE-FABP7 (P) or knockout FABP7 (Q) BMDMs (n=3). (R) qPCR analysis of M2 relative genes in OE-FABP7 BMDMs combined with DGAT1 inhibitor (n=3). DHA：docosahexaenoic acid; FAO: fatty acid oxidation. Data are presented as the mean ± SEM. p values were determined by two-way ANOVA (A, E and N–Q), one-way ANOVA (C, M and R) or unpaired two-tailed Student's t-test (H–L). * $p < 0.05$, ** $p < 0.01$, *** $p < 0.001$, **** $p < 0.0001$; ns, not significant.

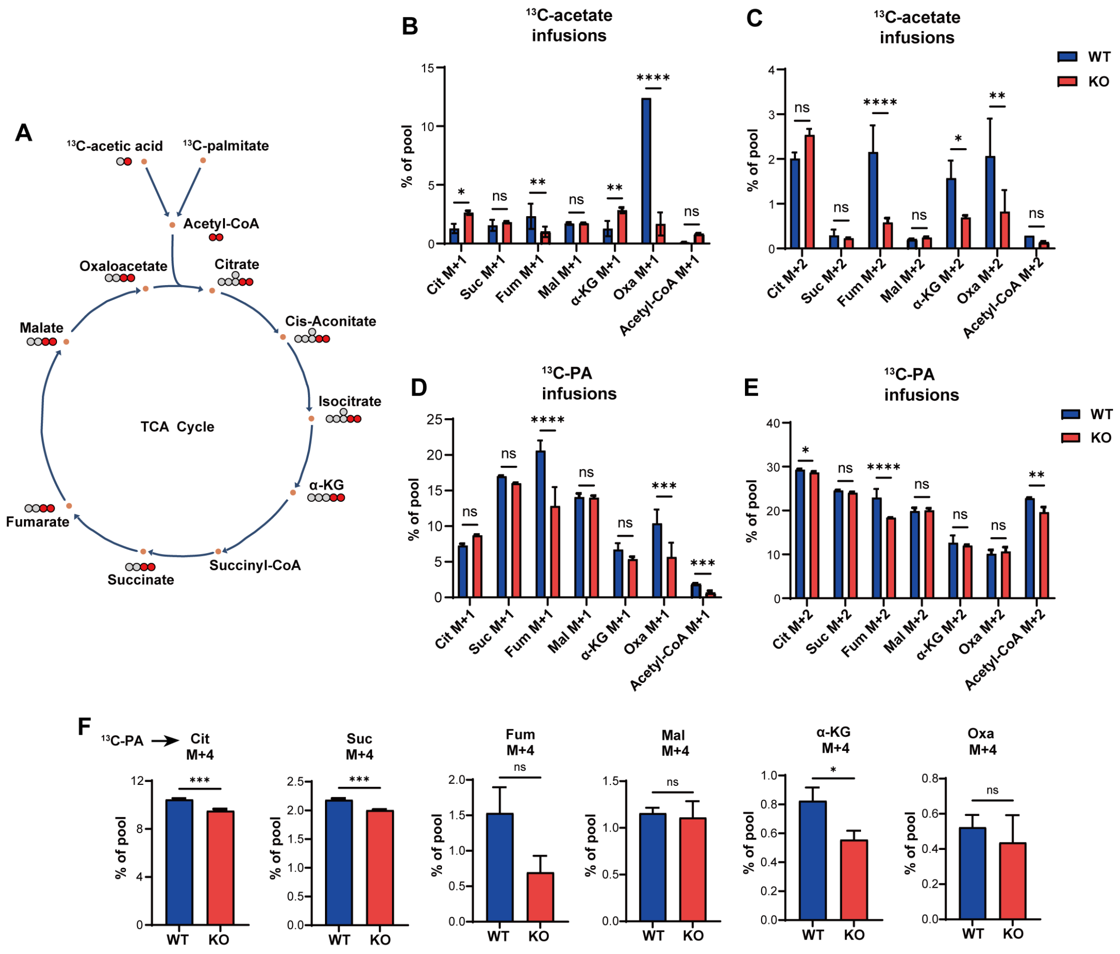

Figure S6. Knockout of FABP7 suppresses lipid as TCA cycle fuel for macrophages.

(A) Schematic depicting ^{13}C–acetate and ^{13}C–palmitate (PA) tracing into WT and FABP7 KO BMDMs metabolites following in vitro infusion. (B–C) Examination of the mass isotopolog distribution (MID) of ^{13}C–acetate labeling into intracellular TCA cycle intermediates in BMDMs isolated from WT and FABP7 KO mice (n=3). M + n isotopologs are plotted as % of pool. (D–F) Examination of the mass isotopolog distribution (MID) of ^{13}C–PA labeling into intracellular TCA cycle intermediates in BMDMs isolated from WT and FABP7 KO mice (n=3). M + n isotopologs are plotted as % of pool. Tricarboxylic acid (TCA), Citrate (Cit), Succinate (Suc), Fumarate (Fum), Malate (Mal), α-ketoglutarate (α-KG), and Oxaloacetate (Oxa). Data are presented as the mean ± SEM. p values were determined by two-way ANOVA (B–E) or unpaired two-tailed Student's t-test (F). * $p < 0.05$, ** $p < 0.01$, *** $p < 0.001$, **** $p < 0.0001$; ns, not significant.

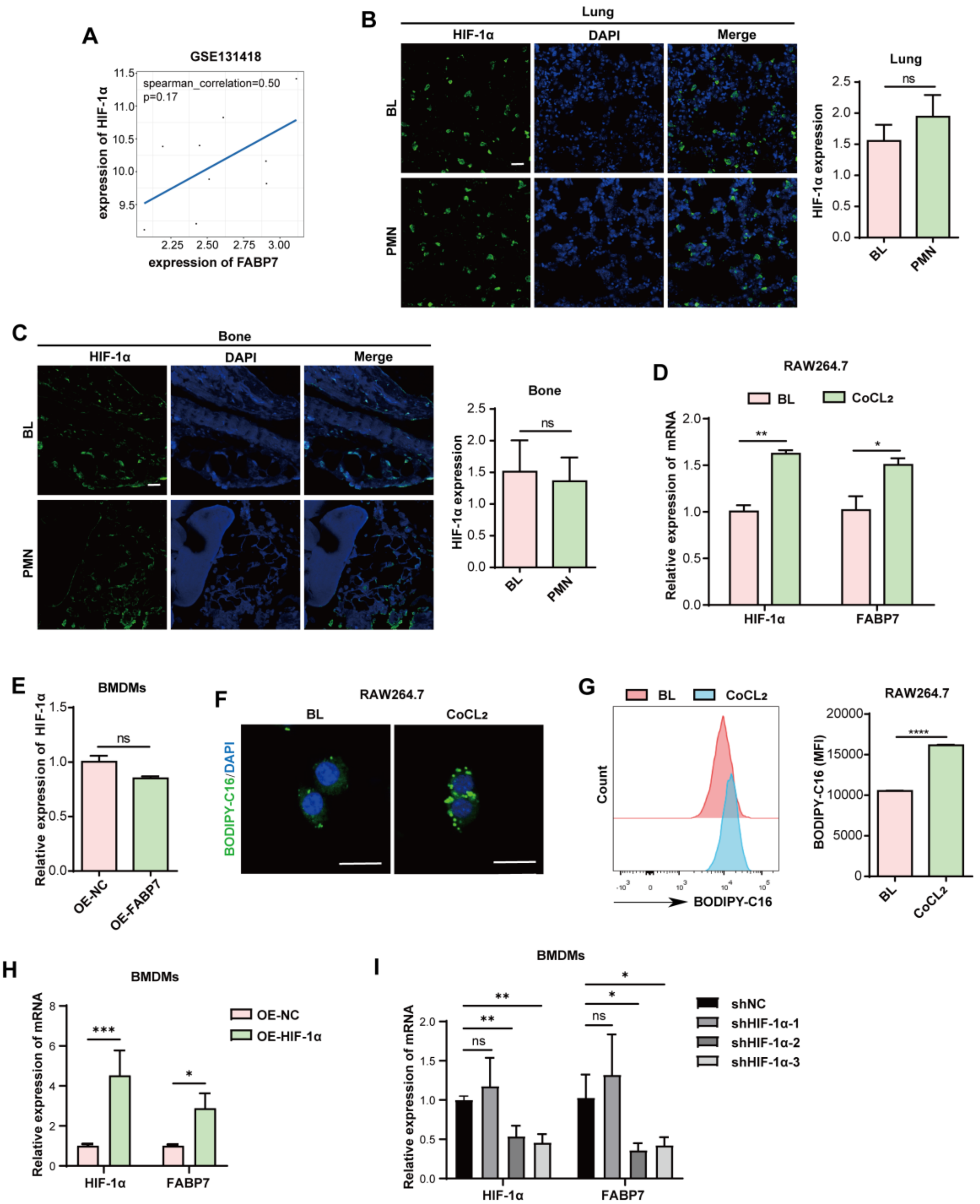

Figure S7. Hypoxia mediates FABP7 expression and lipid accumulation in macrophages.

(A) The correlation of HIF-1α and FABP7 expression analysis of database GSE131418 for human CRC patients. (B–C) Representative images of IF staining of HIF-1α (green) in lungs (B) and bones (C) from mice bearing MC38 in PMN (n=3). Scale bars, 20 μm. (D) qPCR analysis of HIF-1α and FABP7 expression in RAW264.7 cells induced by $CoCL_2$ (n=3). (E) qPCR analysis of HIF-1α expression in OE-FABP7 BMDMs (n=3). (F–G) Fatty acid uptake in RAW264.7 cells induced by

$CoCL_2$ were analyzed by lipid staining (F) and flow cytometry (G, n=3). Scale bars, 10 μm. (H) qPCR analysis of HIF-1α and FABP7 expression in OE- HIF-1α BMDMs (n=3). (I) qPCR analysis of HIF-1α and FABP7 expression in shHIF-1α BMDMs (n=3). Data are presented as the mean ± SEM. p values were determined by two-way ANOVA (D and H–I) and unpaired two-tailed Student's t-test (B–C, E and G). * $p < 0.05$, ** $p < 0.01$, *** $p < 0.001$, **** $p < 0.0001$; ns, not significant.

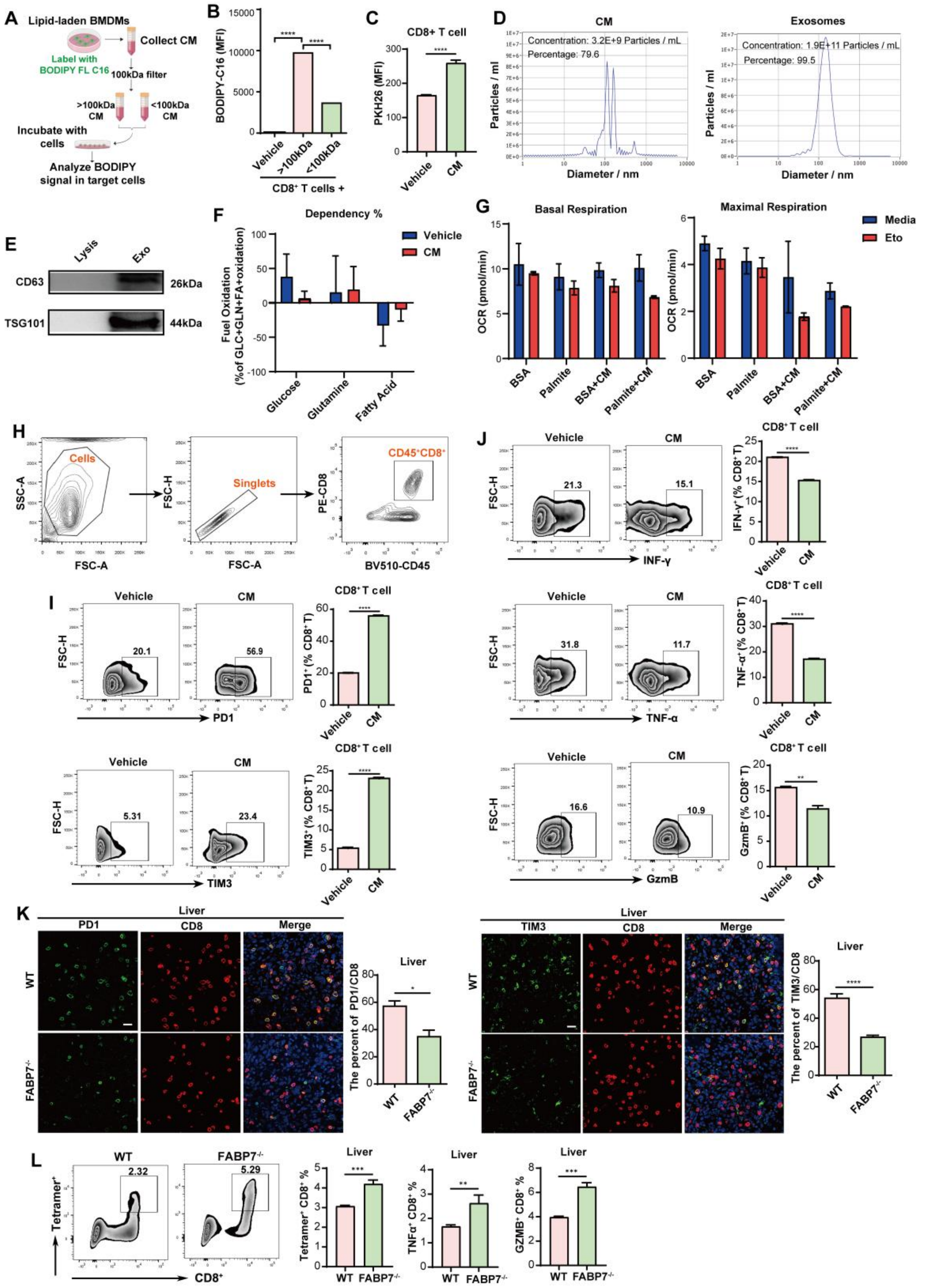

Figure S8. Lipid-laden macrophages transports lipids to CD8$^+$ T cells, leading to CD8$^+$ T cell dysfunction.

(A) As depicted in the schematic, CM was prepared from BODIPY-C16 labeled BMDMs and separated with a 100 kDa filter. (B) Fluorescent lipids incorporated into

$CD8^+$ T cells were detected after incubation with different fractions for 24 h by flow cytometry (n=3). (C) Flow cytometry analysis of PKH26 in $CD8^+$ T cells cultured with CM from PKH26-label BMDMs (n=3). (D) Representative images of NTA of CM before ultracentrifugation and the exosomes isolated from CM after ultracentrifugation. (E) Representative images of Western Blot analysis of CD63 and TSG101 expression of exosomes isolated from CM after ultracentrifugation. (F) Fuel dependency of $CD8^+$ T cells incubated with CM from BODIPY-C16 labeled BMDMs to oxidize glucose, glutamine, and fatty acids of mitochondrial respiration (n=3). (G) Oxygen consumption rate (OCR) of palmitate in $CD8^+$ T cells incubated with CM from BODIPY-C16 labeled BMDMs (n=3). (H) The gating strategy for TIL-$CD8^+$ T cell population is shown. (I) The PD1 and TIM3 expression of $CD8^+$ T cells incubated with CM from BODIPY-C16 labeled BMDMs were determined by flow cytometry (n=3). (J) The indicated cytokines expression of $CD8^+$ T cells incubated with CM from BODIPY-C16 labeled BMDMs were determined by flow cytometry (n=3). (K) IF staining of PD1 or TIM3 (green) expression in TIL-$CD8^+$ T cells (red) in livers of WT and $FABP7^{-/-}$ mice with MC38 metastatic (n=3). Scale bars, 20 μm. (L) Flow cytometry analysis of $Tetramer^+$, $TNFa^+$ and $GzmB^+$ in $CD8^+$ T cells in the liver of WT and $FABP7^{-/-}$ mice bearing OVA-MC38 in MMN (n=3). NTA, nanoparticle tracking analysis. Data are presented as mean ± SEM. p values were determined by one-way ANOVA (B), two-way ANOVA (F–G) or unpaired two-tailed Student's t-test (C and I–L). * $p < 0.05$, ** $p < 0.01$, *** $p < 0.001$, **** $p < 0.0001$; ns, not significant.

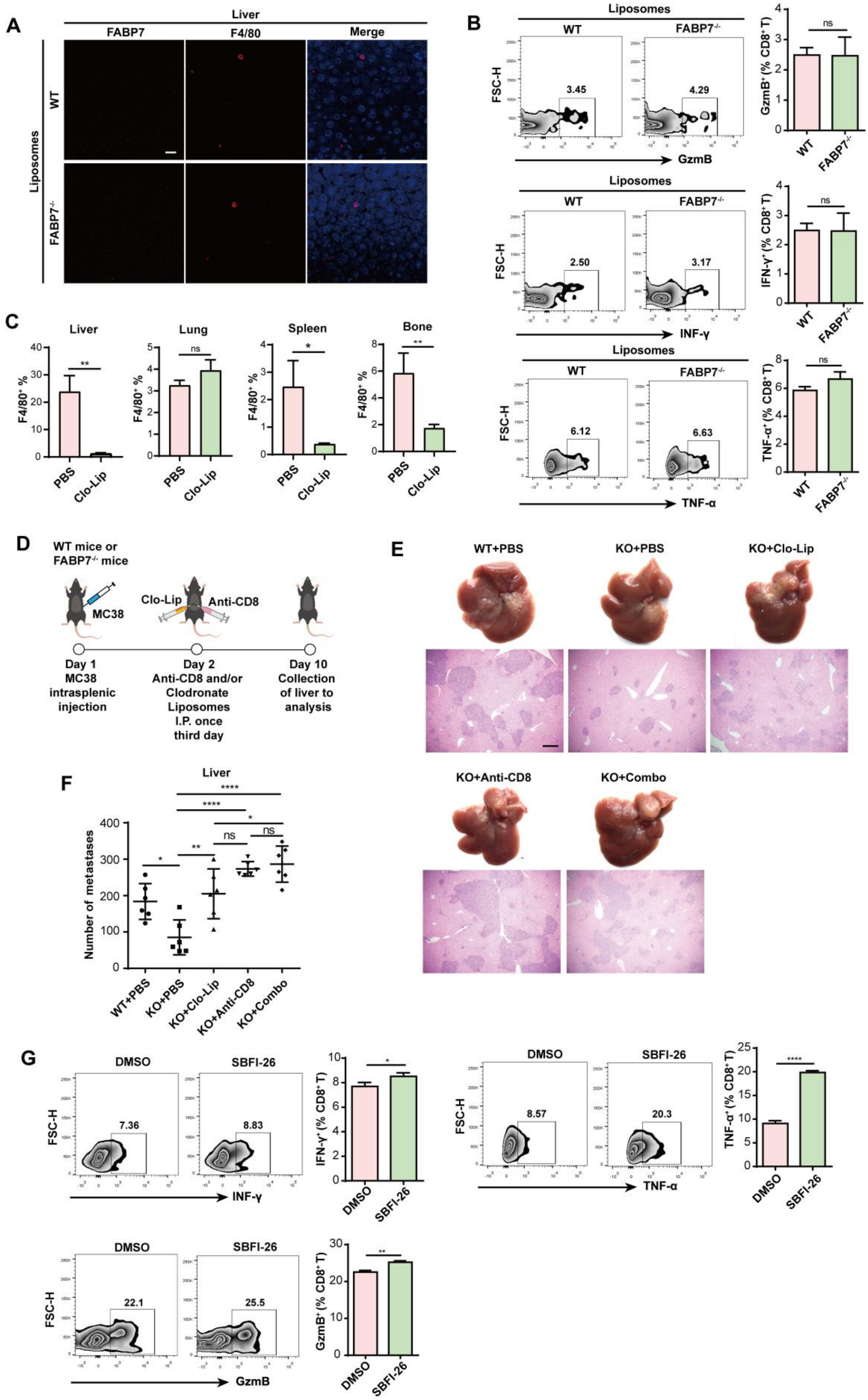

A
Liver
FABP7
F4/80
Merge
Liposomes
WT
FABP7-/-
B
Liposomes
WT
FABP7-/-
FSC-H
3.45
4.29
GzmB
GzmB+ (% CD8+ T)
ns
2.50
3.17
INF-γ
IFN-γ+ (% CD8+ T)
6.12
6.63
TNF-α
TNF-α+ (% CD8+ T)
C
Liver
Lung
Spleen
Bone
F4/80+ %
PBS
Clo-Lip
D
WT mice or FABP7-/- mice
MC38
Clo-Lip
Anti-CD8
Day 1 MC38 intrasplenic injection
Day 2 Anti-CD8 and/or Clodronate Liposomes I.P. once third day
Day 10 Collection of liver to analysis
E
WT+PBS
KO+PBS
KO+Clo-Lip
KO+Anti-CD8
KO+Combo
F
Liver
Number of metastases
WT+PBS
KO+PBS
KO+Clo-Lip
KO+Anti-CD8
KO+Combo
G
DMSO
SBFI-26
FSC-H
7.36
8.83
INF-γ
IFN-γ+ (% CD8+ T)
8.57
20.3
TNF-α
TNF-α+ (% CD8+ T)
22.1
25.5
GzmB
GzmB+ (% CD8+ T)

Figure S9. Clearing macrophages eliminates the effect of FABP7 on the anti-tumor function of CD8 T cells.

(A) Representative images of IF staining of FABP7 (green) expression and F4/80 (red) in livers of WT and FABP7$^{-/-}$ mice bearing MC38 treated with clodronate liposomes. Scale bars, 20 μm. (B) Flow cytometry analysis of IFN-γ, TNF-α and GzmB expression in CD8^{+} T cells in the liver of WT and FABP7$^{-/-}$ mice bearing MC38 treated with clodronate liposomes (n=3). (C) Flow cytometry analysis of macrophages in different tissue (liver, lung, spleen and bone) of mice bearing MC38 treated with clodronate liposomes (n=3). (D–F) As depicted in the schematic (D), the alone or combined effect of Clo-Lip and anti-CD8α in controlling liver metastasis of WT and FABP7$^{-/-}$ mice was determined (E–F, n=6). Scale bars, 200 μm. (G) Flow cytometry analysis of IFN-γ, TNF-α and GzmB expression in CD8^{+} T cells in the liver of WT and FABP7$^{-/-}$ mice bearing MC38 treated with SBFI-26 (n=3). Clo-Lip, clodronate liposomes. Data are presented as mean ± SEM. p values were determined by one-way ANOVA (F) or unpaired two-tailed Student's t-test (B–C and G). * $p < 0.05$, ** $p < 0.01$, **** $p < 0.0001$; ns, not significant.

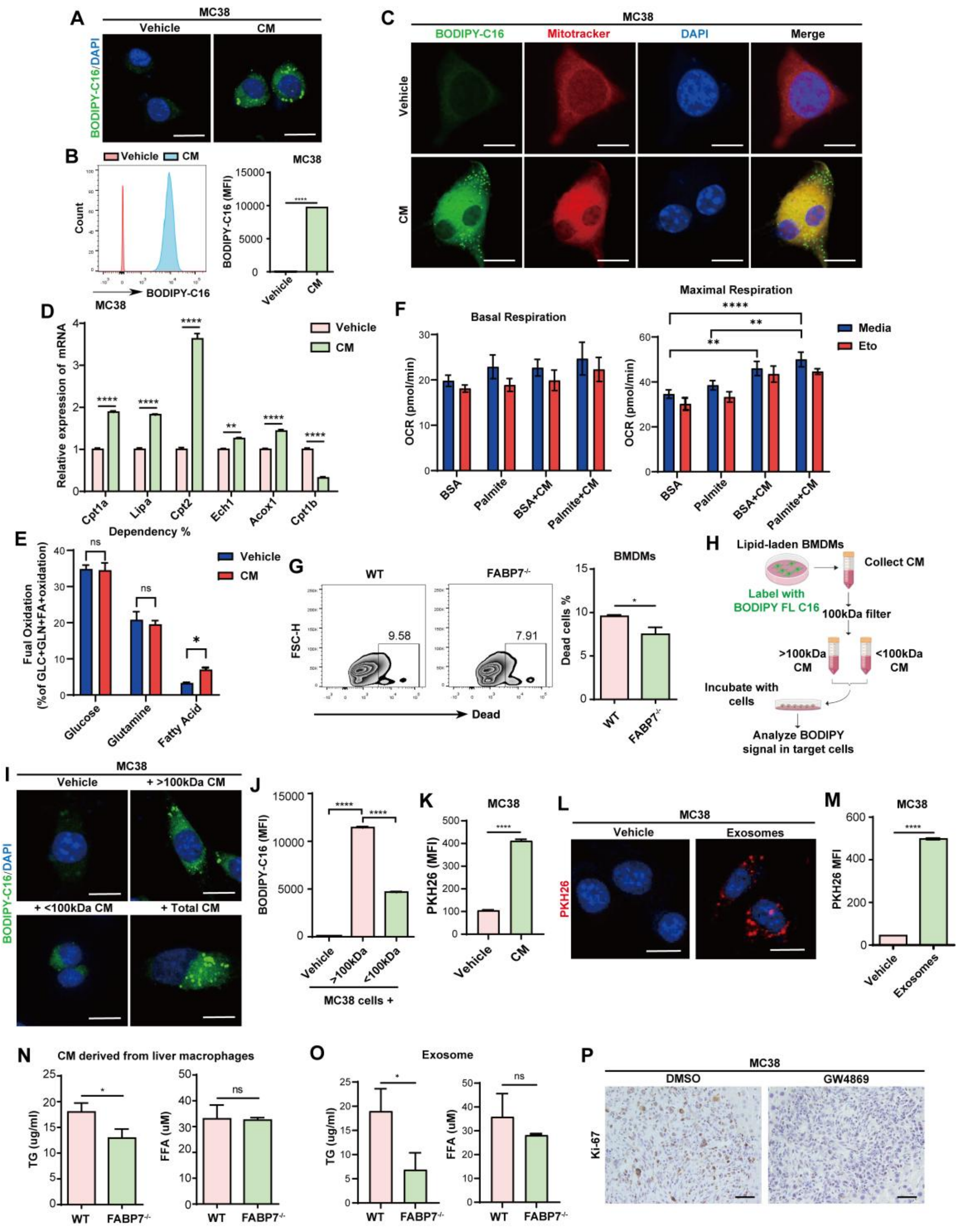

Figure S10. Lipid-laden liver macrophages transport lipids to tumor cells via exosomes.

(A–B) Lipid staining (A) and flow cytometry analysis (B) of lipid contents in MC38 cells incubated with CM from BODIPY-C16 labeled BMDMs (n=3). Scale bars, 10 μm. (C) Representative images of localization of the incorporated lipids (green) with MitoTracker (red) in MC38 cells cultured with CM from BODIPY-C16 labeled

BMDMs. Scale bars, 10 µm. (D) qPCR analysis the expression of the indicated genes in MC38 cells incubated with CM from BODIPY-C16 labeled BMDMs (n=3). (E) Fuel dependency of MC38 cells incubated with CM from BODIPY-C16 labeled BMDMs to oxidize glucose, glutamine, and fatty acids of mitochondrial respiration (n=3). (F) Oxygen consumption rate (OCR) of palmitate in MC38 cells incubated with CM from BODIPY-C16 labeled BMDMs (n=3). (G) Flow cytometry analysis of live/dead cells in WT and FABP7$^{-/-}$ BMDMs cocultured with MC38 cells (n=3). (H) As depicted in the schematic, CM was prepared from BODIPY-C16 labeled BMDMs and separated with a 100 kDa filter. (I–J) Fluorescent lipids incorporated into MC38 cells were detected after incubation with different fractions for 24 h by confocal microscopy (I) and flow cytometry (J, n=3). Scale bars, 10 µm. (K) Flow cytometry analysis of PKH26 in MC38 cells cultured with CM from PKH26-label BMDMs (n=3). (L–M) Lipid staining (L) and flow cytometry analysis (M) of exosome contents in MC38 cells incubated with exosomes isolated from BMDMs (n=3). Scale bars, 10 µm. (N–O) TG and FFA levels detection in CM (N) or exosomes (O) isolated from liver macrophages isolated from WT and FABP7$^{-/-}$ mice bearing MC38 (n=3). (P) Representative images of IHC staining of Ki-67 in livers from mice with MC38 metastases treated with GW4869. TG, triglyceride; FFA, free fatty acid. Data are presented as mean ± SEM. p values were determined by unpaired two-tailed Student’s t-test (B, G, K, and M–O), one-way ANOVA (J) or two-way ANOVA (D–F). * $p < 0.05$, ** $p < 0.01$, **** $p < 0.0001$; ns, not significant.

Supplementary Tables

Table S1. Clinical information of human samples

Variables	Sex	Age	Diagnosis	Group
Patient 1	Female	32	Intrahepatic bile duct stones with biliary dilatation and pericholangitis	Cohort 1
Patient 2	Female	43	chronic cholecystitis	Cohort 1
Patient 3	Female	65	chronic cholecystitis	Cohort 1
Patient 4	Female	55	Xanthogranulomatous cholecystitis	Cohort 1
Patient 5	Male	56	chronic cholecystitis	Cohort 1
Patient 6	Female	51	chronic cholecystitis	Cohort 1
Patient 7	Female	26	chronic cholecystitis	Cohort 1
Patient 8	Male	66	Intrahepatic bile duct stones with biliary dilatation and pericholangitis	Cohort 1
Patient 9	Male	57	chronic cholecystitis	Cohort 1
Patient 10	Male	46	Chronic suppurative cholecystitis	Cohort 1
Patient 11	Female	35	chronic cholecystitis	Cohort 1
Patient 12	Male	60	chronic cholecystitis	Cohort 1
Patient 13	Male	53	chronic cholecystitis	Cohort 1
Patient 14	Male	39	chronic cholecystitis	Cohort 1
Patient 15	Female	34	chronic cholecystitis	Cohort 1
Patient 16	Male	34	chronic cholecystitis	Cohort 1
Patient 17	Male	71	chronic cholecystitis	Cohort 1
Patient 18	Male	56	Traumatic rupture of the liver	Cohort 1
Patient 19	Female	44	Traumatic rupture of the liver	Cohort 1
Patient 20	Male	63	Traumatic rupture of the liver	Cohort 1
Patient 21	Male	70	Traumatic rupture of the liver	Cohort 1
Patient 22	Male	57	Traumatic rupture of the liver	Cohort 1
Patient 23	Male	66	Moderately differentiated adenocarcinoma in the right hemicolon	Cohort 2
Patient 24	Male	63	Moderately differentiated adenocarcinoma of the sigmoid colon	Cohort 2
Patient 25	Female	39	Moderately differentiated adenocarcinoma of the left colon	Cohort 2
Patient 26	Female	42	Mucinous adenocarcinoma of the right hemicolon	Cohort 2
Patient 27	Male	69	Moderately differentiated adenocarcinoma of the left colon	Cohort 2

Patient 28	Male	60	Moderately differentiated adenocarcinoma of the rectum	Cohort 2
Patient 29	Male	72	Mucinous adenocarcinoma of the sigmoid colon	Cohort 2
Patient 30	Male	61	Moderately differentiated adenocarcinoma of the sigmoid colon	Cohort 2
Patient 31	Male	62	Moderately differentiated adenocarcinoma of the left colon	Cohort 2
Patient 32	Male	65	Moderately differentiated adenocarcinoma of the rectum	Cohort 2
Patient 33	Female	32	Moderately differentiated adenocarcinoma in the right hemicolon	Cohort 2
Patient 34	Male	66	Moderately differentiated adenocarcinoma of the rectum	Cohort 2
Patient 35	Male	42	Moderately differentiated adenocarcinoma in the right hemicolon	Cohort 2
Patient 36	Female	57	Moderately differentiated adenocarcinoma in the right hemicolon	Cohort 2
Patient 37	Male	64	Moderately differentiated adenocarcinoma of the rectum	Cohort 2
Patient 38	Male	74	Moderately differentiated adenocarcinoma in the right hemicolon	Cohort 2
Patient 39	Female	61	Moderately differentiated adenocarcinoma in the right hemicolon	Cohort 2
Patient 40	Female	45	Moderately differentiated adenocarcinoma of the sigmoid colon	Cohort 2
Patient 41	Male	57	Well-differentiated adenocarcinoma of the sigmoid colon	Cohort 2
Patient 42	Male	64	Moderately differentiated adenocarcinoma in the right hemicolon	Cohort 2
Patient 43	Male	60	Moderately differentiated adenocarcinoma in the right hemicolon	Cohort 2
Patient 44	Male	83	Moderately differentiated adenocarcinoma in the right hemicolon	Cohort 2

Patient 45	Male	64	Moderately differentiated adenocarcinoma of the sigmoid colon	Cohort 2
Patient 46	Female	42	Well-differentiated adenocarcinoma of the left hemicolon	Cohort 2
Patient 47	Male	43	Poorly differentiated adenocarcinoma of the rectum	Cohort 2
Patient 48	Female	57	Moderately differentiated adenocarcinoma of the sigmoid colon	Cohort 2
Patient 49	Male	50	Liver metastasis from moderately differentiated adenocarcinoma of the rectum	Cohort 3
Patient 50	Male	62	Liver metastasis from moderately differentiated adenocarcinoma in the left colon	Cohort 3
Patient 51	Female	38	Liver metastasis from moderately differentiated adenocarcinoma of the rectum	Cohort 3
Patient 52	Male	39	Liver metastasis from moderately differentiated adenocarcinoma of the rectum	Cohort 3
Patient 53	Male	53	Liver metastasis from moderately differentiated adenocarcinoma of the colon	Cohort 3
Patient 54	Female	62	Liver metastasis from adenocarcinoma of the colon	Cohort 3
Patient 55	Male	76	Colorectal adenocarcinoma with liver metastasis	Cohort 3
Patient 56	Male	67	Liver metastasis from moderately differentiated adenocarcinoma of the colon	Cohort 3
Patient 57	Female	42	Hepatic metastasis of colonic carcinoma	Cohort 3
Patient 58	Male	71	Liver metastasis from moderately differentiated adenocarcinoma in the right colon	Cohort 3
Patient 59	Male	72	Liver metastasis from moderately differentiated adenocarcinoma in the right colon	Cohort 3
Patient 60	Male	57	Liver metastasis from moderately differentiated adenocarcinoma of the sigmoid colon	Cohort 3
Patient 61	Male	50	Liver metastasis from	Cohort 3

			adenocarcinoma of the colon	
Patient 62	Male	37	Hepatic metastasis of colonic carcinoma	Cohort 3
Patient 63	Male	62	Liver metastasis from moderately differentiated adenocarcinoma of the sigmoid colon	Cohort 3
Patient 64	Male	70	Liver metastasis from moderately differentiated adenocarcinoma of the rectum	Cohort 3
Patient 65	Female	57	Well-differentiated adenocarcinoma of the sigmoid colon with liver metastasis	Cohort 3
Patient 66	Female	63	Liver metastasis from moderately differentiated adenocarcinoma in the right colon	Cohort 3
Patient 67	Male	48	Liver metastasis from moderately differentiated adenocarcinoma of the sigmoid colon	Cohort 3
Patient 68	Male	45	Liver metastasis from adenocarcinoma of the colon	Cohort 3
Patient 69	Female	40	Liver metastasis from adenocarcinoma of the colon	Cohort 3
Patient 70	Male	57	Liver metastasis from moderately differentiated adenocarcinoma of the sigmoid colon	Cohort 3
Patient 71	Male	77	Liver metastasis from moderately differentiated adenocarcinoma of the sigmoid colon	Cohort 3
Patient 72	Male	77	Liver metastasis from adenocarcinoma of the colon	Cohort 3
Patient 73	Female	64	Liver metastasis from adenocarcinoma of the colon	Cohort 3
Patient 74	Female	59	Liver metastasis from moderately differentiated adenocarcinoma in the left colon	Cohort 3
Patient 75	Female	61	Liver metastasis from moderately differentiated adenocarcinoma in the right colon	Cohort 3
Patient 76	Male	52	Liver metastasis from moderately differentiated adenocarcinoma of the colon	Cohort 3
Patient 77	Male	75	Liver metastasis from adenocarcinoma of the colon	Cohort 3
Patient 78	Female	67	Liver metastasis from moderately	Cohort 3

			differentiated adenocarcinoma of the sigmoid colon	
Patient 79	Female	57	Pancreatic adenocarcinoma with liver metastasis	Cohort 4
Patient 80	Male	49	Pancreatic adenocarcinoma with liver metastasis	Cohort 4
Patient 81	Male	62	Pancreatic adenocarcinoma with liver metastasis	Cohort 4
Patient 82	Male	61	Pancreatic adenocarcinoma with liver metastasis	Cohort 4
Patient 83	Male	46	Pancreatic adenocarcinoma with liver metastasis	Cohort 4
Patient 84	Male	48	Pancreatic adenocarcinoma with liver metastasis	Cohort 4
Patient 85	Male	48	Pancreatic adenocarcinoma with liver metastasis	Cohort 4
Patient 86	Male	63	Pancreatic adenocarcinoma with liver metastasis	Cohort 4
Patient 87	Male	72	Pancreatic adenocarcinoma with liver metastasis	Cohort 4
Patient 88	Male	57	Pancreatic adenocarcinoma with liver metastasis	Cohort 4
Patient 89	Female	55	Pancreatic adenocarcinoma with liver metastasis	Cohort 4
Patient 90	Male	65	Pancreatic adenocarcinoma with liver metastasis	Cohort 4
Patient 91	Female	77	Pancreatic adenocarcinoma with liver metastasis	Cohort 4
Patient 92	Male	65	Liver metastasis from poorly differentiated adenocarcinoma of the head of the pancreas	Cohort 4
Patient 93	Female	56	Pancreatic adenocarcinoma with liver metastasis	Cohort 4

Table S2. Fatty acids in the liver of tumor-free and tumor-carrying mice.

Fatty acids	Tumor-free		Tumor-carrying	
	Content (ng/ml)	Rate (%)	Content (ng/ml)	Rate (%)
Butyric acid (C4:0)	2.0677	0.039	2.2425	0.041
Hexanoic acid (C6:0)	1.3452	0.025	1.3264	0.025
Octanoic acid (C8:0)	0.7762	0.015	0.7513	0.014
Decanoic acid (C10:0)	0.649	0.012	0.6308	0.012
Undecanoic acid (C11:0)	0.9098	0.017	1.392	0.026
Dodecanoic acid (C12:0)	0.5848	0.011	0.6236	0.012
Tridecylic acid (C13:0)	1.1296	0.021	1.2289	0.023
Myristic acid (C14:0)	5.0367	0.094	3.4236	0.063
Pentadecanoic acid (C15:0)	881.5179	16.488	719.1402	13.289
Palmitic acid (C16:0)	301.0736	5.631	246.1701	4.549
Palmitoleic acid (C16:1)	43.7185	0.818	480.3848	8.877
Heptadecanoic acid (C17:0)	1045.7781	19.560	849.6701	15.701
Stearic acid (C18:0)	261.2376	4.886	290.1486	5.362
Oleic acid (C18:1, n9)	155.9015	2.916	91.341	1.688
Linoleic acid (C18:2, n6)	339.0561	6.342	296.8797	5.486
Leinoleic acid (C18:2)	311.5792	5.828	272.7506	5.040
γ-Linolenic acid (C18:3, n6)	244.1462	4.567	214.5031	3.964
Arachidic acid (C20:0)	2.1996	0.041	2.1702	0.040
Eicosenoic acid (C20:1)	9.9637	0.186	7.34	0.136
Arachidonic acid (C20:4, n6)	483.5009	9.043	453.7444	8.385
Eicosapentaenoic acid (C20:5, n3)	27.7706	0.519	40.2096	0.743
Heneicosanoic acid (C21:0)	4.1958	0.078	23.6074	0.436
Behenic acid (C22:0)	3.7992	0.071	3.7394	0.069
Erucic acid (C22:1, n9)	13.2646	0.248	5.7025	0.105
Tricosanoic acid (C23:0)	5.3006	0.099	5.6561	0.105
Tetracosanoic acid (C24:0)	9.8545	0.184	121.6314	2.248
Docosahexaenoic acid (C22:6, n3)	382.6456	7.157	523.6572	9.677

Table S3. Sequence information in this study.

Primer Name	Sequence (5' to 3')	Assay
Fabp7-F	TCAGGATTGTGATGTGTGAACTGG	Genotyping
Fabp7-R1	TGCTATAAGAAAGGCTACTGTATGG	Genotyping
Fabp7-R2	CACTCCCTTCTCCAAAAGTTACAG	Genotyping
Fabp7-F	CCTCATGATAGAAGTTAGGCCTTGA	CHIP-qPCR
Fabp7-R	ACCCCAGACATCCTCAAGAGT	CHIP-qPCR
Fabp7-F	GCACATTCAAGAACACGGAGA	qPCR
Fabp7-R	CACATCACCAAAAGTAAGGGTCA	qPCR
β-actin-F	GTCCCAGACATCAGGGAGTAA	qPCR
β-actin-R	TCGGATACTTCAGCGTCAGGA	qPCR
Cd206-F	CTCTGTTCAGCTATTGGACGC	qPCR
Cd206-R	CGGAATTTCTGGGATTCAGCTTC	qPCR
Arg1-F	CTCCAAGCCAAAGTCCTTAGAG	qPCR
Arg1-R	AGGAGCTGTCATTAGGGACATC	qPCR
Il10-F	GCTCTTACTGACTGGCATGAG	qPCR
Il10-R	CGCAGCTCTAGGAGCATGTG	qPCR
Tnf-α-F	CCCTCACACTCAGATCATCTTCT	qPCR
Tnf-α-R	GCTACGACGTGGGCTACAG	qPCR
Il1β-F	GCAACTGTTCCTGAACTCAACT	qPCR
Il1β-R	ATCTTTTGGGGTCCGTCAACT	qPCR
Il6-F	TAGTCCTTCCTACCCCAATTTCC	qPCR
Il6-R	TTGGTCCTTAGCCACTCCTTC	qPCR
Dgat1-F	TCCGTCCAGGGTGGTAGTG	qPCR
Dgat1-R	TGAACAAAGAATCTTGCAGA GA	qPCR
Dgat2-F	GCGCTACTTCCGAGACTACTT	qPCR
Dgat2-R	GGGCCTTATGCCAGGAAACT	qPCR
Atgl-F	CCAACACCAGCATCCAGTTCA	qPCR
Atgl-R	CTCCAGCGGCAGAGTATAGGG	qPCR
Hsl-F	GGTGACACTCGCAGAAGACA	qPCR

Hsl-R	GATGGCAGGTGTGAACTGGA	qPCR
Mgl-F	CGGACTTCCAAGTTTTTGTCAGA	qPCR
Mgl-R	GCAGCCACTAGGATGGAGATG	qPCR
Lipa-F	TGACTGACTAGCAAGCGTCCACAA	qPCR
Lipa-R	ATTACCTCCACACAAGGGCCAGAA	qPCR
Cpt1a-F	CTCCGCCTGAGCCATGAAG	qPCR
Cpt1a-R	CACCAGTGATGATGCCATTCT	qPCR
Cpt1b-F	GCACACCAGGCAGTAGCTTT	qPCR
Cpt1b-R	CAGGAGTTGATTCCAGACAGGTA	qPCR
Cpt2-F	CCTCATGATAGAAGTTAGGCCTTG	qPCR
Cpt2-R	GAAGGAACAAAGCGGATGAG	qPCR
Ech1-F	CAGATCAAGGAGGTAGATATGGG	qPCR
Ech1-R	ACAGAATGGTCTCGGGAGTAGA	qPCR
Acox1-F	CAGCACTGGTCTCCGTCATG	qPCR
Acox1-R	CTCCGGACTACCATCCAAGATG	qPCR
Hif-1α-F	ACCTTCATCGGAAACTCCAAAG	qPCR
Hif-1α-R	ACTGTTAGGCTCAGGTGAACT	qPCR
Pgc1a-F	TATGGAGTGACATAGAGTGTGCT	qPCR
Pgc1a-R	CCACTTCAATCCACCCAGAAAG	qPCR
Pparα-F	AGAGCCCCATCTGTCCTCTC	qPCR
Pparα-R	ACTGGTAGTCTGCAAAACCAAA	qPCR
Pparβ-F	TCCATCGTCAACAAAGACGGG	qPCR
Pparβ-R	ACTTGGGCTCAATGATGTCAC	qPCR
Pparγ-F	GGAAGACCACTCGCATTCCTT	qPCR
Pparγ-R	GTAATCAGCAACCATTGGGTCA	qPCR

Table S4: Antibodies and reagents used in the present study.

Antibodies or reagents	Manufacturer	Cat. number
**Antibodies**		
BV510 anti-mouse CD45	Biolegend	Cat# 103137
PE/Cy7 anti-mouse F4/80	Biolegend	Cat# 123113
FITC anti-mouse CD163	eBioscience	Cat# 11-1631-82
PE anti-mouse CD8a	Biolegend	Cat# 162303
APC anti-mouse IFN-γ	Biolegend	Cat# 505809
PE/Cy7 anti-mouse TNF-α	Biolegend	Cat# 506324
FITC anti-mouse GranzymeB	Biolegend	Cat# 372206
Brilliant Violet 510™ anti-mouse CD45	Biolegend	Cat# 103138
Pacific Blue™ anti-mouse CD45.1	Biolegend	Cat# 110722
Brilliant Violet 510™ anti-mouse CD45.2	Biolegend	Cat# 109838
Alexa Fluor® 647 anti-mouse CLEC4F	Biolegend	Cat# 156804
Brilliant Violet 421™ anti-mouse CD68	Biolegend	Cat# 137017
APC/Cyanine7 anti-mouse CX3CR1	Biolegend	Cat# 149047
Brilliant Violet 605™ anti-mouse CD86	Biolegend	Cat# 105037
APC anti-mouse CD80	Biolegend	Cat# 104714
FITC anti-mouse IL10	eBioscience	Cat# 11-7101-82
APC- anti-mouse CD25	Biolegend	Cat# 101909
PE anti-mouse CD278	Biolegend	Cat# 117405
anti-CD3	BD Pharmingen	Cat# 553057
anti-CD28	BD Pharmingen	Cat# 553294
anti-BLBP/FABP7	Abcam	Cat# ab32423
anti-BLBP/FABP7 for human IHC	Sino Biological	Cat# 12577-T60
anti-F4/80	Cell Signaling Technology	Cat# 70076
anti-CD163	Proteintech	Cat# 16646-1-AP
anti-αSMA	Proteintech	Cat# 14395-1-AP
anti-Ly6G	BD Pharmingen	Cat# 551459
anti-CD8a	Cell Signaling Technology	Cat# 98941

anti-CD4	Cell Signaling Technology	Cat# 25229
anti-CD19	Cell Signaling Technology	Cat# 90176
anti-Granzyme B	Cell Signaling Technology	Cat# 44153
anti-CD68	Proteintech	Cat# 66231-2-Ig
anti-CD86	Cell Signaling Technology	Cat# 91882S
anti-HIF1α	Abcam	Cat# ab51608
anti-ADRP	Proteintech	Cat# 15294-1-AP
anti-Beta Actin	Proteintech	Cat# 81115-1-RR
anti-TSG101	Immunoway	Cat# B6001
anti-CD63	ABClonal	Cat# A5271
goat anti-rabbit IgG/Alexa Fluor	Bioss	Cat# [illegible]
goat anti-mouse IgG/Alexa Fluor	Bioss	Cat# [illegible]
goat anti-rabbit IgG	ZSGB-BIO	Cat# PV-6001
goat anti-mouse IgG	ZSGB-BIO	Cat# PV-6002
Goat anti-Rabbit IgG (H + L) Secondary Antibody, HRP	Fdbio science	Cat# FDR007
Goat anti-Mouse IgG (H + L) Secondary Antibody, HRP	Fdbio science	Cat# FDM007
InVivo anti-mouse CD8α	Biocell	Cat# BE0061
**Reagents**		
LIVE/DEAD™ Fixable Dead Cell Stain	Thermo Fisher Scientific	Cat# L34966
4% paraformaldehyde, PFA	Biosharp	Cat# BL539A
DMEM	Gibco	Cat#
fetal bovine serum	Gibco	Cat# 10099-141
Penicillin/streptomycin	NCM	Cat# C100C5
CoCL2	Sigma	Cat# 232696
SBFI-26	MedChemExpress	Cat#
DHA	MedChemExpress	Cat# HY-B2167
A922500	Selleck	Cat# S2674
GW4869	Selleck	Cat# S7609
clodronate liposomes	Yeason	Cat# 40337ES10
GM-CSF	PeproTech	Cat# 315-02
LPS	MedChemExpress	Cat# HY-D1056

IFN-γ	PeproTech	Cat# 315-05
IL-4	PeproTech	Cat# 214-14
IL-10	PeproTech	Cat# 210-10
Hank's buffered salt solution	Gibco	Cat#
ethylene diamine tetraacetic acid (EDTA)	LEAGENE	Cat# R00020
Collagenase, type IV	Sigma	Cat# V900893
DNAse I	Sigma	Cat# DN25
Hyaluronidase	Sigma	Cat# H3506
BODIPY493/503	Invitrogen	Cat# D3922
BODIPY-C16	Invitrogen	Cat# D3821
Cell Activation Cocktail (with Brefeldin A)	Biolegend	Cat# 423303
DAPI	Beyotime	Cat# C1006
RIPA buffer	Fdbio science	Cat# FD008
TRIzol reagent	Takara	Cat# 9108
SYBR Green PCR Master Mix	Takara	Cat# RR820A
Gelred	Tsingke	Cat# TSJ003
DL2000 DNA Marker	Tsingke	Cat# TSJ011-100
MitoTracker® Red CMXRos	Yeason	Cat# 40741ES50
PKH26	BestBio	Cat# BB-441125
[1-$^{13}C_1$] acetic acid	MedChemExpress	Cat# HY-Y0319S1
[$^{13}C_{16}$] palmitate	MedChemExpress	Cat# HY-N0830S6
Seahorse XF DMEM medium, pH	Agilent	Cat# 103575-100
XF 1.0 M Glucose Solution	Agilent	Cat# 103577-100
XF 100 mM Pyruvate Solution	Agilent	Cat# 103578-100
XF 200 mM Glutamine Solution	Agilent	Cat# 103579-100
EasySep™ Mouse F4/80 Positive Selection Kit	STEMCELL	Cat# 100-0617
EasySep™ Mouse CD8+ T Cell Isolation Kit	STEMCELL	Cat# 19853A
Mouse Direct PCR Kit (For Genotyping)	Bimake	Cat# B40015
Seahorse XF Cell Mitochondrial Pressure Test Kit	Agilent	Cat# 103015-100
Seahorse XF Glycolysis Pressure Test Kit	Agilent	Cat# 103020-100
Seahorse XF LCFA Oxidation Stress Test Kit	Agilent	Cat# 103672-100

Hoechst 33342	Solarbio	Cat# C0031
Bradford Protein Assay	KeyGEN BioTECH	Cat# KGB2101-100
Supelco 37 component FAME mix	Sigma	Cat# CRM47885
ECL Western blotting detection reagents	Fdbio science	Cat# FD8030
Free Fatty Acid Fluorometric Assay Kit	Cayman	Cat# 700310
Triglyceride Colorimetric Assay Kit	Cayman	Cat# 10010303
SimpleChIP® Enzymatic Chromatin IP Kit	Cell Signaling Technology	Cat# 9003
Luciferase reporter assay	Transgen	Cat# FR201
fixation/permeabilization solution kit folgiplug	BD Pharmingen	Cat# 554714
FcR block reagent	MEDICAL & BIOLOGICAL LABORATORIES	Cat# PN MTG-001
H-2K^b OVA Tetramer -SIINFEKL-APC	MEDICAL & BIOLOGICAL LABORATORIES	Cat# TS-5001-2C
